# The concept of biophotonic signaling in the human body and brain: rationale, problems and directions

**DOI:** 10.3389/fnsys.2025.1597329

**Published:** 2025-06-23

**Authors:** Ganna Nevoit, Kristina Poderiene, Maksim Potyazhenko, Ozar Mintser, Gediminas Jarusevicius, Alfonsas Vainoras

**Affiliations:** ^1^Laboratory for Automatization of Cardiovascular Investigations, Cardiology Institute, Lithuanian University of Health Sciences, Kaunas, Lithuania; ^2^Department of Health and Rehabilitation, Lithuanian Sports University Institute of Sports Science and Innovation, Kaunas, Lithuania; ^3^Department of Internal Medicine and Emergency Medicine, Poltava State Medical University, Poltava, Ukraine; ^4^Department of Fundamental Disciplines and Informatics, Shupyk National Healthcare University of Ukraine, Kyiv, Ukraine

**Keywords:** biophoton, ultra-weak photon emission, electromagnetic signaling, biophoton signaling, cellular communication

## Abstract

This perspective piece presents the concept of the role and mechanisms of cells’ electromagnetic communication. These data deepen the scientific understanding of the fundamental aspects of the phenomenon of human life. A promising model of biophoton signaling as a scientific tool for further developing of biophotonics of the human body is substantiated.

## 1 Introduction

Further study of the fundamental aspects of the essence of the phenomenon of biological life of the human organism continues to be a pressing problem of modern science. What mechanisms unite the multicellular human body into a single functional whole? What mechanisms ensure the complex process of precise, simultaneous, and coordinated implementation of genetic information from deoxyribonucleic acid (DNA) molecules? What mechanisms provide instant coordination of many metabolic reactions that co-occur in all living body cells? What mechanisms provide higher mental activity for a person? These and many other similar questions have motivated scientific interest in the problem of cell communication. Initially, science discovered the phenomenon of chemical signaling through molecular interactions (Dutta Banik and Medler, 2022; Lee et al., 2023). Signal transduction, only through signaling molecules, second messengers, and receptors, cannot answer all scientific questions. For example, chemical signaling models cannot explain the mechanism of protein folding and answer the questions outlined at the beginning of the article. The situation has changed now due to the understanding that, along with chemical signaling in the cell and the human body, there is electromagnetic signaling. Scientists began discussing the possibility of electromagnetic interaction between living cells at the beginning of the 20th century after the results of experiments by the Russian and Soviet scientist Gurwitsch (1911, 1923, 1924) and Gurwitsch and Gurwitsch (1960). However, the complexity of the methodology of Gurvich’s experiments, the absence of appropriate measuring instruments for recording electromagnetic interaction, and a theoretical biophysical foundation of knowledge did not allow the science of that time to advance in understanding these issues. Despite many diverse studies, the Biological Field Theory created by Gurwitsch and Gurwitsch (1960), Beloussov et al. (2007), and Nevoit et al. (2024a) was criticized and forgotten for a time. A scientific breakthrough in this field of knowledge occurred in the 1970s and was associated with a series of scientific studies by the German biophysicist F. A. Popp. Thanks to the development of new equipment for recording photons, Popp could study the emission of photons from living biological objects. As the founder of the International Institute of Biophysics (Düsseldorf, Germany), Popp united nineteen research institutions in thirteen countries around the world into a scientific consortium. This made it possible to collect a large layer of fundamentally new information about electromagnetic radiation emitted by living biological cells (Nevoit et al., 2024a). Popp was the first to introduce the concept of “biophoton” (from the Greek “βίος”—life and “Φως”—light, power) into science and created the basis for today’s concepts of electromagnetic cellular communication (Popp, 1976; Chang et al., 1998a, b; Nevoit et al., 2024a). Popp’s research findings identified DNA as the primary source of biophotons (Bischof, 1995). This confirmed the previously put forward ideas that biophotons are formed in the cell. However, for a long time, the radiation of biophotons and the phenomenon of ultra-weak photon emission (UPE) from living biological cells and organisms were considered a by-product of biochemical reactions (Nevoit et al., 2024a). Over the past few decades, the phenomenon of UPE has become a well-studied biophysical phenomenon (Calcerrada and García-Ruiz, 2019; Chai et al., 2022; Sordillo and Sordillo, 2020; Benfatto et al., 2023; Du et al., 2023; Mould et al., 2024; Erboz et al., 2025). The general scientific progress of physics, the development of quantum physics and knowledge related to the Electromagnetic Field Theory, made it possible to rethink the accumulated scientific material on the properties and parameters of UPE. The coherence of the energy of UPE and the properties of solitons became the basis for understanding the scientific fact that this electromagnetic phenomenon is one of the important mechanisms of cellular electromagnetic communication. Understanding this fundamentally changes the vector of scientific views on the phenomenology of the life of cells and multicellular organisms, including the human body, brain, and mind. This is the “big gate” to a new era of future scientific understanding of the fundamental aspects of the essence of the phenomenon of human biological life, to a new scientific paradigm. For modern scientists, this is a new scientific challenge. This is because there is already a large layer of fundamentally new scientific knowledge about the phenomenon of biophoton radiation, but there is no complete systemic biological understanding of it. There is currently no biological theory in science that would logically explain and systematize the role of biophoton emission in cellular metabolism and the phenomenology of the life of a cell and all cells of the human body. Therefore, the aim of this perspective piece was to summarize existing ideas about the communicative role of biophotons in the functioning of cells and the entire human body, and to present them as a working concept of biophoton signaling. This concept will be a starting point for further understanding of the mechanisms of electromagnetic signaling in the human body, and will become the basis for further scientific discussions and continued development of an important scientific direction—biophotonics of the human body (Van Wijk and Shen, 2005).

This perspective piece includes two preliminary sections: brief reviews of modern scientific data on biophotons as energy carriers, information in the human body, and their biological role. Next comes the central section, “The Concept of Biophoton Signaling,” which results from the authors’ theoretical research and summarizes the existing scientific concepts. The author’s vision of the practical significance of the proposed working concept of biophoton signaling is described in the section “Discussion, Problems and Prospects.”

## 2 Biophotons are carriers of energy and information in the human body

Biophoton signaling is a complex scientific view that explains the role and mechanisms of biophotons’ participation in transmitting information at all levels of the hierarchical organization of the human body (cell, tissue, organ, organ system, whole organism). The concept is based on biophotons’ ability to transfer energy and information. What modern scientific data confirms this?

### 2.1 Concept 1: photon is the basis of structural and energetic interaction in the human body

According to the Standard Model, the photon is a gauge boson that mediates the electromagnetic interaction between fermions, the constituent parts of atomic nuclei. The photon is a fundamental electromagnetic field particle and its quantum particle (Wells, 2020; Paganini, 2023; Hübsch, 2023). According to the laws of classical physics, the photon exhibits duality and is both a massless particle and a wave of the visible spectrum of radiation, i.e., light. Photons have no rest mass, no electric charge, do not interact with each other, and always move at the speed of light. Charged particles, such as electrons, can emit and absorb photons. Thus, electromagnetic interaction in nature is carried out exclusively by photon mechanisms: photons cause the transmission of electromagnetic energy between elementary particles with a charge. The study of this is carried out by the section of physics called “photonics” (Saleh and Teich, 2007; Van Wijk and Shen, 2005).

What does this mean for the functioning of the human body? Man is an integral part of nature, and its universal mechanisms are also valid for the functioning of his body. We come to two main conclusions by extrapolating these data to the human body. First, photons participate in the organization of atoms at the quantum level. Therefore, they participate in structuring the human body as matter (Ho, 2003). Secondly, photons ensure the flow of all electromagnetic processes at the quantum level in the human body. This is so because life is an electromagnetic phenomenon of activation of metabolic processes, and the chemistry of molecules is only a secondary derivative phenomenon of electromagnetic quantum parameters of atomic structures. At the present scientific stage, this is conceptualized in a series of scientific publications on the Magnetoelectrochemical Theory of Metabolism and Life (Mintser et al., 2020, 2021, 2022, 2023; Nevoit et al., 2024b). Therefore, the interaction of light/photons and living matter of the human body is a significant problem of fundamental science, which is studied by the section of photonics and biophysics – “biophotonics” (Sterenborg et al., 2011).

Now, thanks to the progress of quantum physics, it has become clear that light/photons are in an even more complex electromagnetic interaction with the material structures of the human body than the data presented above. The above-mentioned properties of photons are characteristic of them only in a medium that does not have a crystal lattice. When propagating in crystalline structures, photons acquire structure, mass, dipole moments, and the ability to interact with each other, collide, and fly apart. In crystals, photons acquire properties comparable to those of a liquid. This happens because a quantum of light/photon in a semiconductor crystal excites an electron that has absorbed this quantum. Fusing a photon and an electron creates a new material object, an “exciton.” This electron-exciton can radiate a photon back and return to its original state, and another electron can absorb the emitted photon. This creates a chain of photon absorption and emission events. Thus, in crystalline structures, photons can be in two fundamentally different states: in the state of a light quantum or the form of a material object – an exciton. This phenomenon was first described in 1958 by J. Hopfield in his article on exciton polaritons (Hopfield, 1958) or, in other words, on quanta of “liquid light.” Since 1957, there has been a narrow field of physics called “polaritonics” that studies the properties of “liquid light”/photons in crystalline structures. Polaritonics is a field of innovation and discovery (Grosso et al., 2024).

Extrapolating the knowledge of polaritonics to the human body, it becomes evident that photons manifest themselves as “liquid light” in the human body. This is because the human body is a unique combination of liquid crystal structures with semiconductor properties. Firstly, water makes up 45 to 90% of the human body composition depending on age (Laja García et al., 2019; Lu et al., 2023). At the same time, a significant part of the water in the body of a living person is in the state of liquid energy-intensive crystals in the form of a spiral 31/21 (Bulienkov and Zheligovskaya, 2006, 2013; Nevoit et al., 2022a). This is why the water of the cytoplasm and intercellular fluid does not have the properties of a simple solvent and exhibits so-called biological anomalies (Köpf et al., 1996). Secondly, the lipid components of the membranes are also in a state of liquid crystals (Nevoit et al., 2022b). Together with a layer of proteins, they form complex structures similar to capacitors, can accumulate an electric charge (Nevoit et al., 2022b). Moreover, membrane structures comprise over 20% of the cell’s composition (Cooper, 2000) and include mitochondria, the cell’s primary “energy, synthetic centers” (Pizzorno, 2014; Nevoit et al., 2024c). Thirdly, connective tissue makes up about 85% of body mass (Potekhina, 2015) and has semiconductor properties. The ability of connective tissue in the collagen protein model to transmit electromagnetic energy through biopolymer chains has been proven (Davydov, 1973, 1974, 1977, 1982; Giraud-Guille, 1989; Blanco-Fernández et al., 2023; Adwan et al., 2024). Proteins act as crystalline semiconductors, and the deformation of connective tissue elements creates piezoelectricity. Every cell is connected to every other cell at any moment, and they communicate (Oschman, 2007, 2009). This determines the unique properties of selective electromagnetic conductivity of the human body at all hierarchical levels of its organization. Thus, the third conclusion is that human body tissues can exhibit the properties of superconductors, functioning within the physiological temperature and instantly transmitting, without loss, electromagnetic energy in the form of photons in their unique state of “liquid light”.

### 2.2 Concept 2: photon is the basis of information interaction in the human body

Everything a person sees, they can see only thanks to photons of light. Of course, for a person, photons are the primary source of visual information about the Universe and the world around us. However, when science says that photon is the basis of informational cellular interaction, it means that biophoton mechanisms provide the transfer of information about metabolic processes in the cell, between cells, in organ tissues, and the human body as a whole. Biophoton mechanisms are an important component of cellular electromagnetic signaling *in vivo* (Paolis et al., 2024; Tong, 2024). Why is this so? This is explained by the fact that a photon can carry information (Saleh and Teich, 2007; Mehra, 2021; Kenneth, 2014). It is well known that the properties of photons to transmit information have found practical implementation in many technical solutions for radio communications, television, compact disc players, computer disk drives, ultra-fast personal computers based on the use of heterostructure semiconductors, pagers, and cellular communications. Photons allow for the achievement of high operating speed, low energy consumption of photonic devices, and the implementation of a quantum communication line, among others. Photons are the fastest carriers of quantum information, and spin memory can reliably store data over a relatively long period. Entangled “spin-photon” pairs are an ideal solution for implementing quantum networks that provide fast and secure transmission of information over long distances (Saleh and Teich, 2007; Liang et al., 2018). Therefore, it will not be surprising that nature could create such a unique quantum system in the human body and the human brain.

How do biophotons transmit information in the human body? The answer to this question will be presented in several stages during the disclosure of the review topic. Basic ideas about the mechanisms of these processes are associated with the features of the formation and transport of electrons and photons along the chains of biopolymer molecules of the membrane structures of human body cells (Graovac et al., 1977). The generally accepted concept is that in the human body, electrons are transferred by the ATP molecule to the biopolymer, move along its chain, and, passing from one reaction participant to another, support various endoergic biochemical reactions (Graovac et al., 1977). However, in the second half of the 20th century, it became clear that the energy transfer model by *π*-electrons, which passed to biopolymers from ATP, could not explain the high actual rate of the process. This was called the “crisis in biophysics” and was discussed at the International Union of Biophysicists in New York (1973, USA) (Mintser et al., 2021). This question received a theoretical solution in discovering a new energy state as a “standing wave”/Davydov soliton (Bolterauer, 1990; Dauxois and Peyrard, 2006; Christiansen and Scott, 2013). It has now been scientifically proven that in the human body, biophotonic mechanisms of informational cellular interaction are realized through the transformation by biopolymers of the incoherent energy of the ATP molecule into the coherent energy of a special type of elongated waves/wave packets – solitons/“standing waves” (Gall, 2010; Mintser et al., 2021). This is a soliton model of energy transfer along a biopolymer chain. A soliton is an organization of electromagnetic energy/biophotons in the form of a separate structurally stable wave in a nonlinear medium with dispersion, which is what living biological systems are. The stationarity of the soliton structure in a medium without losses and energy inflow is maintained due to the balance between the action of the nonlinear medium. This leads to an enlargement (compression) of the wave profile and dispersion, and this ensures the preservation of the shape, the speed of movement of photons, and enables photons to carry information (Brizhik et al., 2009; Mintser et al., 2021; Marin, 2022). The soliton mechanism of energy transfer is the basis of energy and information exchange at the quantum and atomic-molecular levels of the human body structure.

A quantum-mechanical examination of the essence of the electron transfer mechanism in biopolymers shows that solitons are energy quanta that transfer along the molecular chain an amount of energy equal to the energy of the longitudinal vibration of the C=O bond. Solitons are formed during the self-localization of energy in oscillatory processes on various anharmonics of molecular structures, which in their “energetic essence” and in the values of quantum energies are completely identical to photons of the electromagnetic field in the frequency range from ~10^14^ Hz and below. At the same time, this range of low energies is biologically significant, manifested in the active response of living organisms to the external influence of an electromagnetic field of any origin in the range of these frequencies. The soliton mechanism of energy transfer is a universal process in the human body because the energy from the ATP molecule can be transformed into a soliton in any polymer protein *in vivo*. Soliton, as a physical solution to the problem of energy transfer along biopolymer chains, is correct for any protein-ATP system (Davydov, 1973, 1974, 1977, 1982; Nevoit et al., 2022b, 2025a, 2025b; Mintser et al., 2021).

According to modern science, quantum communication is the most efficient. Quantum information is recorded in qubits. Qubits have an arbitrary quantum superposition, not just two basis states. Therefore, the qubit has access to an infinite number of states that fill a two-dimensional space. At the same time, the use of quantum entanglement of several particles can provide an ultra-secure protocol for transmitting information (Paul et al., 2002; Saleh and Teich, 2007; Howe et al., 2023). Photons are perfect natural candidates for the physical implementation of qubits (Shi et al., 2022; Saleh and Teich, 2007). Photons have two basic states—these are two mutually orthogonal polarizations of the photon. Standard optical elements allow for the manipulation of information. The transmission of quantum information encoded in the polarization of light has long been realized both via optical fiber and in the open air over a distance of over 100 km (Saleh and Teich, 2007; Krenn et al., 2016). Also characteristic of photons is the orbital angular momentum or, conventionally speaking, their “twist” relative to the direction of propagation. This phenomenon has been known for a long time and is used to encode information. The twist of photons determines their even greater information capacity because it has an infinite number of available states. This allows for additional multiplexing in the optical communication line (Rossi et al., 2009; Forbesa and Nape, 2019; Saleh and Teich, 2007). Therefore, the photon is the basis of information interaction in the human body, created by nature. Systematic representation and understanding of their biological role in the human body are categorically important for the further development of the biomedical scientific direction.

## 3 The biological role of biophotons in the human body

The correctness of the concepts that photons are the basis of structural, energetic, and informational interaction in the human body is confirmed by established scientific data on their biological role in it. The biological role of biophotons can be simplified as follows.

### 3.1 Biophotonic mechanisms are a universal biophysical phenomenon of life activity for all cells *in vivo*

Scientific research led Popp to the understanding that light is produced inside the cell of a biological organism. This was a provocative and revolutionary idea for that time, but Popp scientifically proved it (Bischof, 1995; Nevoit et al., 2024a). Biophoton emission or UPE is a universal optical phenomenon of electromagnetic radiation for most living biological systems in the spectral range from 200 to 800 nm with a constant rate from several photons per cell per day to several hundred photons per organism per day (Popp et al., 1992, 2002; Bischof, 1995; Popp and Beloussov, 2003; Beloussov et al., 2007; Van Wijk, 2014; Kobayashi et al., 2016; Nevoit et al., 2023). Human vision cannot detect UPE: UPE is 1 E^6^ s^−1^ cm^−2^, and threshold of human vision is 1 E^6^ s^−1^ cm^−2^. UPE has low intensity and higher energy compared to thermal or conventional chemical activation. The energy range of UPE is 1.67–3.41 eV. In the spectral range <700 nm, UPE is 1 E^10^ higher than the statistical Boltzmann distribution and has a value of ~10^−1^ E^4^ photons per s^−1^ cm^−2^ (Popp et al., 1984, 1992, 2002; Popp and Beloussov, 2003; van Wijk et al., 2005; Van Wijk et al., 2006, 2020; Beloussov et al., 2007; Tinsley et al., 2016; Kobayashi et al., 2016; Calcerrada and García-Ruiz, 2019; Kobayashi and Inaba, 2000). UPE is a spatiotemporal manifestation of the biological electromagnetic field energy of a living cell *in vivo*. The coherent properties of this field are biophoton signaling/electromagnetic intercellular signaling (Popp et al., 1984, 1992; Bischof, 1995; Beloussov and Volodyaev, 2013; Popp and Beloussov, 2003; Calcerrada and García-Ruiz, 2019; Kobayashi and Inaba, 2000). In the 21st century, the conceptualization of the role of biophotons in the phenomenology of biological life and the functioning of the human body was carried out (Nevoit et al., 2023).

### 3.2 Biophotonic mechanisms of DNA functioning

According to many scientists (Popp et al., 1984; Pietruszka and Marzec, 2024; Nevoit et al., 2025a), biophotonic mechanisms form the basis of biological life and ensure the functioning of DNA. A model of life regulation by UPE in living organisms and a mechanism of UPE participation in the implementation of genetic information have been developed (Popp et al., 1984; Popp and Beloussov, 2003; Nevoit et al., 2023).

As noted, biophotonic mechanisms are realized in solid-state crystalline structures by forming excitons/exciton polaritons. The DNA molecule is an excited duplex/exciplex system. DNA receives electromagnetic energy in the form of photons and accumulates it. In DNA, photons are efficiently stored between two DNA strands due to the peculiarities of its spatial conformation (Nevoit et al., 2023). DNA has an information density of 1-10^9^ higher than any technical solution. Photons are in the DNA in a Bose-Einstein condensate state (Popp et al., 1984; Popp, 2003; Yip and Madl, 2006; Burgos et al., 2017). In essence, this is an electromagnetic coherent cellular-biological state, in which exciton polaritons/“liquid light” condensation occurs at room temperature. In this case, photons are captured in a “cryo-trap,” compacted and “frozen” in time. This ensures the stability of the DNA molecule: photons of the same frequency and phase are aligned. There is scientific opinion that 97.98% of inactive human DNA, together with the “frozen” energy of biophotons, plays a key role in organizing the 2.02% of genetically expressed DNA (Bischof, 1995; Yip and Madl, 2006).

This confirms the ideas of G. Fröhlich about the biophotonic mechanism of DNA action, which were presented to science in the 50s of the twentieth century (Popp, 2006; Hyland, 2000; Popp and Beloussov, 2003). DNA unwinding during replication causes activation of biophoton emission (Popp, 2005; Popp et al., 1992). The spiral-shaped genetic material in DNA functions as a biological laser (Sitko, 2012; Mintser et al., 2021; Nevoit et al., 2023). Biophotons can be generated due to nucleotides’ luminescent properties during their cooperative behavior in DNA and the constant supply of energy through metabolic processes (Popp, 2005; Popp et al., 1992). In simplified terms, the biophysical mechanism of this can be represented as follows: if the frequency of the oscillatory charge is high and approaches the optical part of the electromagnetic field spectrum, then the generated electromagnetic waves begin to exhibit corpuscular properties and form particles of light – biophotons (Nevoit et al., 2023). The correctness of this is confirmed by the fact that *in situ* at least 75% of biophotonic activity comes from the DNA of cells (Popp, 2006; Popp et al., 1984; Van Wijk, 2001; Scaletta et al., 2001; Levin, 2014, 2021; Nevoit et al., 2023). Stacking bases in DNA provides suitable conditions for the emission of biophotons (Popp, 2005, 2006; Popp et al., 1984; Nevoit et al., 2023).

### 3.3 Biophotonic mechanisms for the implementation of cellular communication, morphogenesis and superposition of cells in tissues and organs

UPE is a registered result of the spatio-temporal manifestation of the energy of cells’ electromagnetic/biophotonic field. At the same time, the superposition of various modes in the optical range of electromagnetic radiation provides a spatially precise resolution of the intensity pattern of “standing waves”/solitons, which carry information (Mintser et al., 2021). The coherence of this electromagnetic field enables this electromagnetic energy to carry information from DNA and to be a communicative mechanism of electromagnetic signaling. The conjugation of the electromagnetic fields of cells leads to their coherent interaction in tissues, organs and in the body. Thus, biophotons are a key tool for intercellular and transcellular communication (Bischof, 1995; Van Wijk, 2001; Levin, 2021; Nevoit et al., 2023).

Emission of biophotons from DNA molecules forms an electromagnetic information field inside and around cells. This is somewhat reminiscent of the information environment of a Wi-Fi zone indoors. At the same time, the DNA molecules perform the “function of routers that distribute the information signal” in cells. Cell membranes are located in the nodal planes of the interference pattern of this electromagnetic field (Mintser et al., 2021). Distribution of energy outside the cellular space serves as a means of communication and interaction in the regulatory processes of the cell cycle. Collagen fibers and the microtubule system act as a “fiber-optic” network that transmits, stores and transmits the biophotonic signal in the cell (Ho, 2003; Yip and Madl, 2006; Nevoit et al., 2023). This forms a constant electromagnetic information field, which receives information content from biophoton signals from DNA molecules. Thus, due to the constant presence in this field, and each cell biopolymer, each structural and functional part of the cell have continuous access to the current genetic information about the metabolic processes necessary for morphogenesis. Due to constant presence in a common conjugate electromagnetic field from many cells, the cells of a certain body locus receive information about their superposition and coordinate the general and specific functions of tissue and organs.

### 3.4 Biophotonic mechanisms of regulation of metabolism and cell activity

The emission of biophotons from DNA molecules and the coherent electromagnetic information field of the cell created on this basis controls chemical reactions in its cytoplasm, provides resonant frequency-wave interactions between cells, is a guiding force for molecules and accurately launches more than 100·10^3^ chemical reactions per second (Van Wijk, 2001; Van Wijk et al., 2020; Nevoit et al., 2023). This is the basis of the deterministic self-organization of molecules in cells and the solution to the mechanisms of protein folding, etc. In addition to the emission of biophotons by DNA molecules, the cytoplasm provides part of the biophotonic activity due to the activity of microtubules involved in the multiplication of biophotonic radiation emanating from the cell nucleus. Microtubules and contact communication connections conduct biophotonic impulses into the extracellular matrix and to neighboring cells (Yip and Madl, 2006: Nevoit et al., 2023). Also, the adhesive forces between the cells connect them into functional blocks, making it possible to form a resonator system for long-wave photons as well. The death of a cell from such a functional block of cells leads to a violation of its overall resonant frequency. This change leads to photon emission and initiates the cellular regeneration process (Bischof, 1995; Levin, 2014). A link has been demonstrated between UPE levels and reactive oxygen species in tissues of living biological systems (Van Wijk et al., 2013; Yip and Madl, 2006). Biophotonic mechanisms are involved in cell aging processes (Niggli, 2014). UPE is a diagnostic indicator of oxidative stress (Burgos et al., 2017).

### 3.5 Biophotonic mechanisms of visual perception

The biological role of biophotonic mechanisms is to form of visual perception, transmit signals from the retina to the brain, and form of visual images in the brain.

The primary function of the eyes is the visual perception of light. The retina is transparent to photons. Photons are perceived in the last layer of the retina, absorbed by photoreceptors, causing action potentials transmitted to the brain (Salari et al., 2012; Yanoff and Duker, 2009).

Biophotonic mechanisms are involved in signal transmission and are used to amplify very weak stimuli. The retinal nervous system has time constants of the order of 10·10^2^ s, which do not correspond to the actual speed of visual perception when one phosphodiesterase molecule is activated after a photon is absorbed. Amplification, amplification occurs at the initial stage, when single-photon excited rhodopsin switches to excitation of at least 500 transducin molecules within 1 ms. Models of visual perception mechanisms involving biophotonic processes continue to be developed (Bischof, 1995; Ho, 2003; Yip and Madl, 2006; Kobayashi et al., 2016). Retinal discrete dark noise has the same velocity as the UPE velocity of photoreceptor cells (Devaraj et al., 1991; Salari et al., 2015).

The mechanisms of visual image formation in the brain continue to be studied. Biophotonic mechanisms are involved in forming specific visual phenomena phosphene, negative afterimage, and retinal discrete dark noise (Tang and Dai, 2014a). Neurons contain many light-sensitive molecules (porphyrin rings, pyridine rings, lipid chromophores and aromatic amino acids, etc.) (Karu, 1999; Kato et al., 1981; Mazhul and Shcherbin, 1999; Thar and Kühl, 2004), which can respond to biophoton signals. Excessive UPE retention in visual areas of the brain may manifest as phosphene lights in our consciousness (Bokkon, 2008). The vision of bright light in NDEs may be caused by UPEs simultaneously generated in multiple areas of the visual system at that moment (Bókkon et al., 2010; Bokkon and Salari, 2012).

### 3.6 Biophotonic mechanisms of brain activity

The brain generates UPE, as proven by Isojima et al. (1995), Kobayashi et al. (1999a, 1999b), Bókkon et al. (2010), and Bókkon et al. (2010).

There is a connection between the intensity of UPE and the level of brain activity and its metabolic state. This confirms the universality of the participation of biophoton mechanisms in the vital processes of brain cells. For example, UPE from neurons has a direct correlation with neuronal activity, with the level of cerebral energy metabolism, electroencephalogram parameters, with the speed of cerebral blood flow, and oxidative processes (Isojima et al., 1995; Kobayashi et al., 1999a, 1999b). UPE increased after induction of cerebral hypoxia and throughout all hypoxic and posthypoxic (reperfusion) states (Imaizumi et al., 1984; Suzuki et al., 1985). UPE was enhanced by depolarization of the hippocampal region with high potassium concentration and was reduced by suppression of neuronal activity with tetrodotoxin (Isojima et al., 1995). UPE intensity is related to cerebral blood flow (Kobayashi et al., 1999a, 1999b)—Glutamate Stimulates UPE in the brain (Tang and Dai, 2014b). UPE depends on the processes of depolarization of the neuronal membrane and the entry of calcium ions into cells (Kataoka et al., 2001), etc.

Biophotons are involved in transmitting nerve impulses in the peripheral nervous system. Electrical stimulation of the nerve increased UPE, which completely disappeared after the nerve’s death (Artem’ev et al., 1967; Salari et al., 2016). UPE closely correlates with axonal action potential generation as measured by electroencephalography (Dotta and Persinger, 2011; Dotta et al., 2012).

Do biophoton mechanisms participate in the implementation of higher nervous activity? Nervous activity is associated with the morphological integrity of the brain, with the functional state of neurons, and with the generation and transmission of complex electromagnetic impulses in the consolidated system of brain neurons. Therefore, the answer to this question relates to quantum mechanics, magnetic field theory, and biophoton energy, and information transfer mechanisms. This has been repeatedly mentioned by many scientists (Brixner et al., 2005; Engel et al., 2007; Fenna and Matthews, 1975; Adolphs and Renger, 2006; Lee et al., 2007; Ishizaki and Fleming, 2009) when discussing the role of quantum effects in biology. The results of some experimental and clinical studies indicate a relationship between biophotonic mechanisms and higher nervous activity. The correlation between neuronal metabolic activity and UPE intensity has been postulated as a new mechanism for processing neural information (Tang and Dai, 2014a, 2014b; Esmaeilpour et al., 2020). UPE correlates with neuronal activity (Devaraj et al., 1991; Kobayashi et al., 1999a, 1999b; Cifra and Pospíšil, 2014). Electroencephalography parameters strongly correlate with UPE in the human brain (Kobayashi et al., 1999a, 1999b; van Wijk et al., 2008; Dotta et al., 2012). A weak but significant phase and frequency relationship was established between the alpha rhythm of the brain and UPE (van Wijk et al., 2008). The influence of meditation sessions on the intensity of UPE processes has been established (van Wijk et al., 2005, 2006). Using electroencephalography data to establish a connection between UPE from brain neurons and cognitive functions during subjective visual images (Dotta and Persinger, 2011; Dotta et al., 2012).

Thus, biophoton mechanisms are universal mechanisms for regulating vital activity in all cells of the human body and brain. But how is all this united in the human body? What are the systemic mechanisms, stages and interrelationships of these processes? The answer to this question may be a working model of biophoton signaling, created as an attempt to link everything that is happening into a single system of ideas.

## 4 The biophoton signaling concept

The first stage of the biophoton signaling concept: the formation of coherent electromagnetic energy.

To date, a working model of biophoton signaling has been conceptualized at the level of cells, the intercellular matrix (Nevoit et al., 2025a), and the whole organism (Nevoit et al., 2024d). The concept of biophoton signaling at the cellular level is part of the concept of a biological medical theory that could describe the substrate and mechanisms of transmission of electromagnetic communication at the level of organs, organ systems, and the organism as a whole (Nevoit et al., 2024d). This describes the universal mechanisms of electromagnetic signaling without considering its cellular and tissue specificity. This concept is consistent with existing scientific models of chemical interactions of substances in cells and explains the issues of complex coordination of their metabolic transformation. It complements the existing scientific paradigm of knowledge and does not contradict it.

### 4.1 The first stage of the biophoton signaling concept: the formation of coherent electromagnetic energy

**Figure 1:**
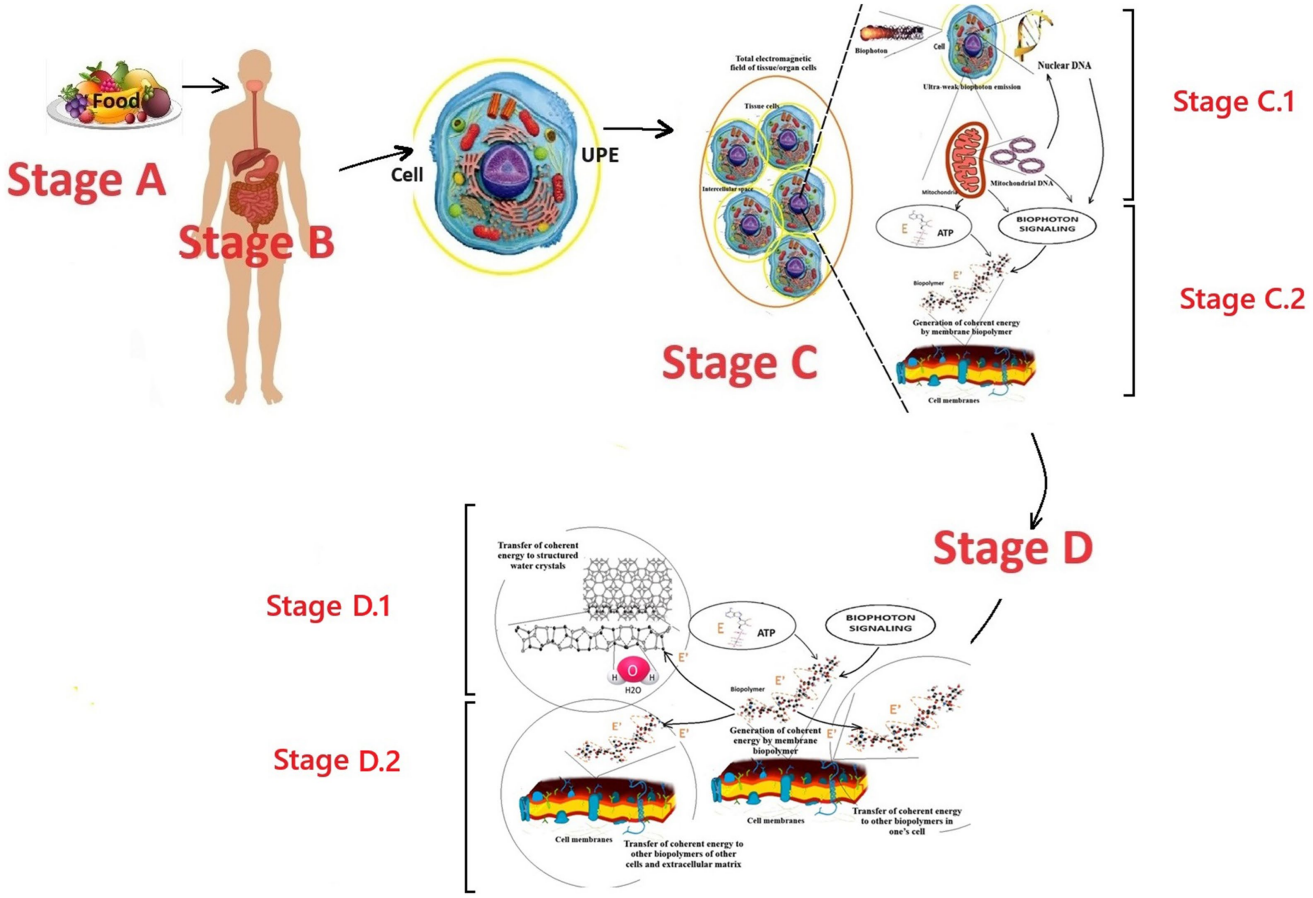
Perspective scheme of the first stage of the biophoton signaling concept model: formation of coherent electromagnetic energy by cell membrane biopolymers. Fragments of Nevoit et al. (2025a, 2025b) were used. E is incoherent energy that comes to biopolymers from the universal chemical carrier—the ATP molecule; E’ is coherent energy that is formed by the oscillatory activity of biopolymers; the yellow circle around the cell is a conditional representation of the UPE phenomenon; the dotted curve is a conditional representation of the biopolymer oscillation processes. Stage A is the entry of energy in the form of food into the human body. Stage B is the digestion of food, assimilation of food substrates, and their entry into the blood and cells of the human body. Stage C corresponds to the cell’s UPE and tissue respiration processes, which lead to the formation of biophotons and a universal energy carrier—the ATP molecule, respectively. Stage C.1 is the formation of an internal electromagnetic information field in a cell due to the emission of biophotons from the nucleus’s DNA molecules and the mitochondria’s DNA. Stage C.2 is the generation of coherent electromagnetic energy by the biopolymers of the cell membranes. Stages C.1 and C.2 co-occur and provide the emergence of a coherent electromagnetic current in the membranes, which carries energy and an information component from the DNA. Stage D.1 is the transition of coherent energy to the liquid crystal structures of water molecules. Stage D.2 is the transition of coherent energy to other biopolymers. Stages D.1 and D.2 co-occur and provide the emergence of a coherent electromagnetic current in the cell membranes, in the cellular and intercellular matrix.

In simplified terms, the first stage can be outlined as follows (Figure 1).

According to the modern scientific paradigm, energy enters the human body through food (stage A). In the gastrointestinal tract, food is digested into food substrates (stage B). Food substrates are absorbed and enter the cells with the blood flow (stage C). In the cell, food substrates are metabolized by mitochondria in the Krebs cycle to form a universal chemical energy carrier—adenosine triphosphate (ATP) molecules. ATP molecules interact with any cell biopolymers, transferring a portion of chemical energy of ≈0.18 eV to the partner molecule and turning into adenosine diphosphate. Then, in the biopolymer, this “chemical” incoherent energy is transformed into electromagnetic energy specific to the biopolymer. This is stage C.2 in the presented model of biophoton signaling.

According to the soliton model of energy transfer along the biopolymer chain (Davydov, 1973, 1974, 1977, 1982), the following is the description. The energy transferred to the biopolymer by the ATP molecule causes its chain excitation and oscillations. The groups involved in the excitation of Amide I, which are capable of longitudinal vibrations only due to the rigidity of the double bond C=O, strain the molecule’s structure within this bond. This local distortion of the lattice is a potential well that localizes the vibrational energy and prevents its dispersion. In such a chain, the vibrational energy of longitudinal C=O oscillators (Amide I), with the help of phonon bonds (bonds of crystal lattice vibrations), acts on neighboring atomic groups and changes the chain structure within the C=O. This change results in the capture of energy by the Amide I group, preventing further energy dispersion. This phenomenon has been called the energy self-channeling effect. The emergence of self-channeling of energy is associated with the violation of rigidity in the biopolymer chain. “Flexible” bonds between peptide groups in the chain C-N, C-C determine the minimum values of its longitudinal elasticity. In contrast, large ones provide for significant interaction between the vibrations of Amide I and phonons. Obtaining a solution for an optical soliton (a soliton that is formed by the absorption of light or another quantum of an electromagnetic field) is the following result of the development of Davydov’s theory, which took into account quantum fluctuations of the equilibrium positions of molecules and their thermal vibrations relative to new equilibrium positions. It has been established that in this case, a quasiparticle moves from the molecular chain, which is an electron with a local deformation surrounding it—an electrosoliton. The stability of the electrosoliton is due to the mutual compensation of the nonlinearity and dispersion effects in the molecular chain. It is most pronounced in soft one-dimensional chains of peptide groups in proteins. Always moving at a speed lower than the speed of longitudinal sound, the electrosoliton does not emit phonons, i.e., does not lose its energy (Davydov, 1973, 1974, 1977, 1982; Nevoit et al., 2022b, 2025a, 2025b; Mintser et al., 2021).

A “soliton flow” is formed, which moves further without loss through biopolymers (Del Giudice et al., 2010) and liquid crystal structures of water (Bulienkov and Zheligovskaya, 2006, 2013), carrying energy and information. Due to this, a coherent electromagnetic current occurs in all molecules of the cell membrane structures and the cell cytoplasm. In the same way, this energy from the membrane structures can pass to the membrane structures of neighboring cells, water liquid-crystalline structures, and other molecules of the intercellular matrix. This is stage D. Where did the cell-specific biologically significant information in this coherent energy flow come from?

According to the presented perspective model of biophoton signaling, this is due to the processes of stage C.1. The emission of biophotons from DNA molecules is the source of this information. The DNA of the nucleus and mitochondria emits biophotons with genetic information and creates an electromagnetic/biophoton field. The corresponding molecules of membrane biopolymers perceive information from this field according to the resonance principle. This phenomenon of information transmission in the form of an electromagnetic field is biophoton signaling. Under the influence of biophoton signaling from DNA, biopolymers form their information component in the electromagnetic energy they generate. This coherent electromagnetic energy of biopolymers becomes the carrier of this information. Thus, at stages D.1 and D.2, this biophoton signaling is further transmitted to water molecules and other biopolymers. This circulation of coherent electromagnetic energy ensures the flow of energy and information from the DNA of the nucleus and mitochondria to each molecule of the cell, “feeds” and “informs” it. This determines the unification mechanism of all cell molecules into a single functional structure, subject to genetic and metabolic coordination from the DNA molecules of the nucleus and mitochondria.

The presented fragment of the first stage of the concept of biophoton signaling is valid for any living cell *in vivo*. However, cells in the human body have different localizations, structures, functions, numbers of mitochondria, and structures of mitochondria, and they obey different programs of realization of their genetic code from their DNA. Accordingly, the qualitative and quantitative characteristics of biophoton signaling are cellular and tissue-specific. Therefore, detailing the mechanisms of biophoton signaling for tissues of different organs will have tissue and organ specificity according to the presented concept.

The specificity of biophoton signaling will determine the number and structural features of mitochondria in each cell type. This is because mitochondria “control” two ways of influencing the biophoton signaling process. Firstly, the density and power of electromagnetic biophoton signaling depend on the number of mitochondria in a cell. The more mitochondria a cell has, the greater the biophoton radiation it can produce. The set of chromosomal apparatus/DNA is initially the same in the nucleus of each somatic cell, but the number of mitochondria is different. The number and structure of mitochondria depend on the cell type and can change under the influence of epigenetic factors. For example, the number of mitochondria decreases with physical inactivity, exposure to toxic agents, etc. (Lane, 2005; Leduc-Gaudet et al., 2021; Nevoit et al., 2024c).

The most significant number of mitochondria in the human body is found in tissue cells with an active level of metabolic processes (Casanova et al., 2023). The leaders in mitochondria content are the cells of striated muscles and neurons of the brain (Bruce and Johnson, 2002): the number of mitochondria in them is about 22% of the dry residue of the cell. Moreover, it is no coincidence that the brain, heart, and muscles generate the most powerful electromagnetic fields. For example, the heart generates the strongest electromagnetic field and typically containing from 5,000 to 7,000 mitochondria in one cardiomyocyte. Mitochondria can occupy up to 40% of the volume of a myocardiocyte (Bruce and Johnson, 2002). The existence of a cause-and-effect relationship between the number of mitochondria in the cells of an organ and the total power of its electromagnetic generation is another confirmation of the correctness of the logic of the fact that the histological features and functions of the tissue influence the cellular specificity of biophoton signaling. Accordingly, different tissues and organs contribute to the overall electromagnetic/biophoton generation in the human body.

### 4.2 The second stage of the biophoton signaling concept: the transmission of coherent electromagnetic energy

The second stage of biophoton signaling is the transmission of coherent electromagnetic energy throughout the body, which is formed by the biopolymers of the cell membranes of the entire human body. Electromagnetic energy is subject to the universal laws of nature. According to the electromagnetic field theory, free charges move in one direction in a conductor, forming an electromagnetic current. In the human body, electromagnetic energy can move through the molecules of biopolymers, the lipid layers of the membrane structures of cells, and connective tissue because most organic molecules are liquid crystals and exhibit semiconductor properties. The circulation of electromagnetic energy is the main condition for the implementation of the phenomenon of biological life at the micro level of the human body structure because all molecules need this specific coherent electromagnetic energy for their existence, vibrations, and obtaining information about metabolic processes (Nevoit et al., 2024d). To implement the phenomenon of life at all structural levels of the human body, electromagnetic energy/biophoton signaling must flow continuously through the molecules.

The transmission of coherent electromagnetic energy/biophoton signaling occurs because each cell, each tissue, and each organ generates and emits specific electromagnetic energy with information from the DNA of its nuclei and mitochondria. This radiative process from all cells forms local quantum electromagnetic fields in all parts of the human body. Electromagnetic fields of different parts of the body must interact with each other according to the universal laws of physics. Energy must be transferred from the zone where it is more to where it is less and create electromagnetic currents—the transmission of coherent electromagnetic energy from one body part to another. Therefore, in the body of a living person, there must be a constant circulation of coherent electromagnetic current and a general coherent electromagnetic field, which is formed due to this movement. This constant circulation of the coherent electromagnetic field inside a living organism/biophoton signaling transfers energy and information from each cell to other cells, uniting them into a single functional whole at the level of the entire organism.

**Figure 2:**
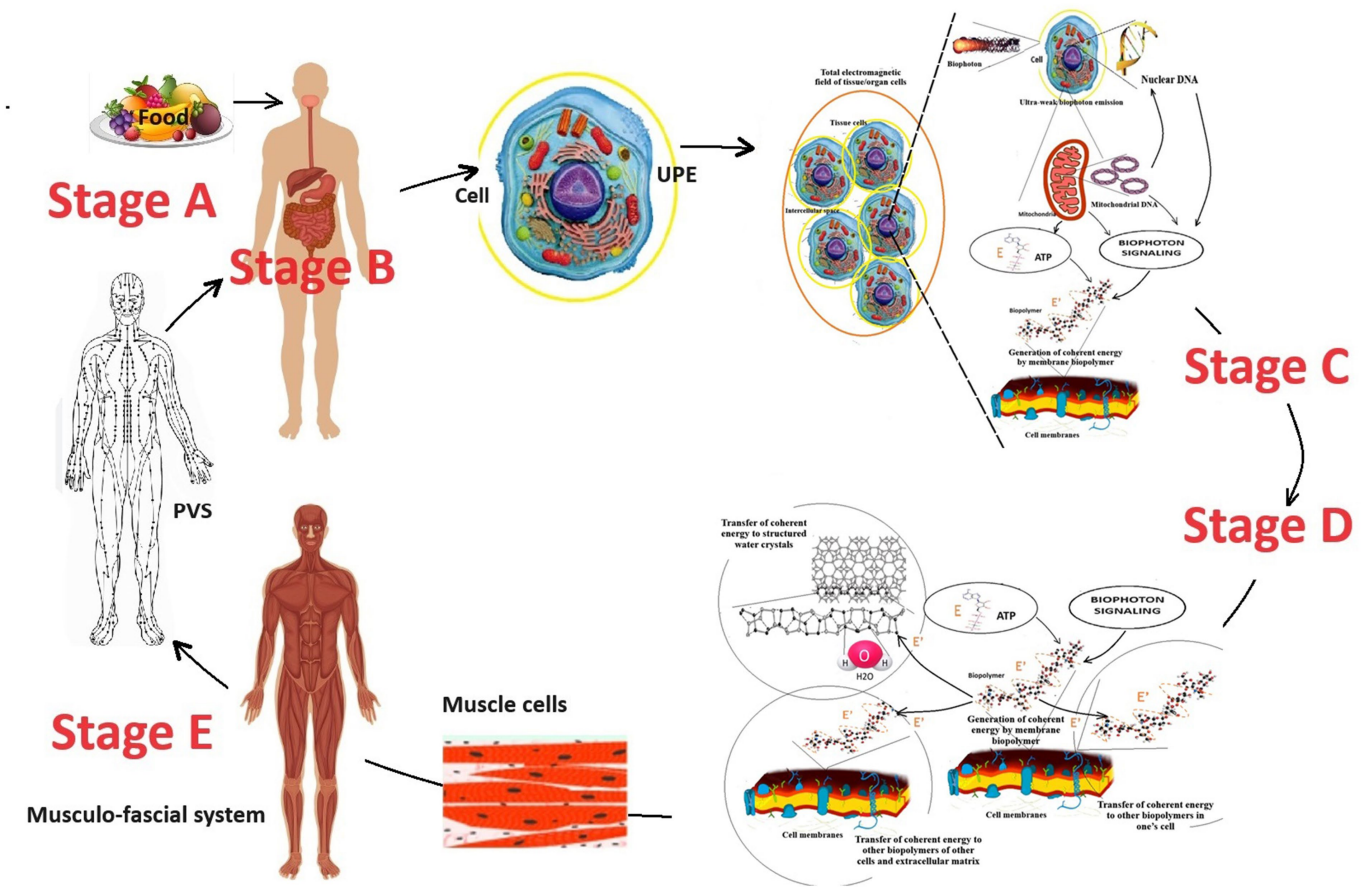
The perspective scheme of energy exchange in the human body considers the working model of the biophoton signaling concept. The figure was created using fragments of illustrations (Nevoit et al., 2024d, 2025a). E is incoherent energy that comes to biopolymers from the universal chemical carrier—the ATP molecule; E’ is coherent energy that is formed by the oscillatory activity of biopolymers; the yellow circle around the cell is a conditional representation of the UPE phenomenon; the dotted curve is a conditional representation of the oscillation processes of biopolymers. Stage A is the entry of energy in the form of food into the human body. Stage B is the digestion of food, assimilation of food substrates, and their entry into the blood and cells of the human body. Stage C corresponds to the cell’s UPE and tissue respiration processes, which lead to the formation of biophotons and a universal energy carrier—the ATP molecule, respectively. Stage D occurs on the biopolymers of membranes of all cells and ensures the transformation of the biochemical energy of the ATP molecule into electromagnetic energy. Stage E is the redistribution and transport of electromagnetic energy from muscles to other parts of the human body through myofascial synkinesis (muscle chains) and PVS.

The developed working model of the second stage of biophoton signaling complements the first stage into a holistic system of energy exchange in the human body (Figure 2).

A key aspect of the second stage of biophoton signaling is understanding the quantum role of muscles as the final stage of metabolism and the primary source of electromagnetic generation in the human body.

According to a systematic analysis of existing scientific data (Nevoit et al., 2024d), it was found that muscle tissue produces the most significant amount of coherent electromagnetic energy. Biopolymers of muscle cell membranes are the primary source of electromagnetic current and biophoton signaling in the human body. This conclusion was made because muscle cells contain the most significant number of mitochondria and should have the largest cell mass in the body of a properly developed person. The brain is also among the leaders in the mitochondria content in one cell. However, the brain is inferior to muscle tissue regarding the body’s content.

**Figure 3:**
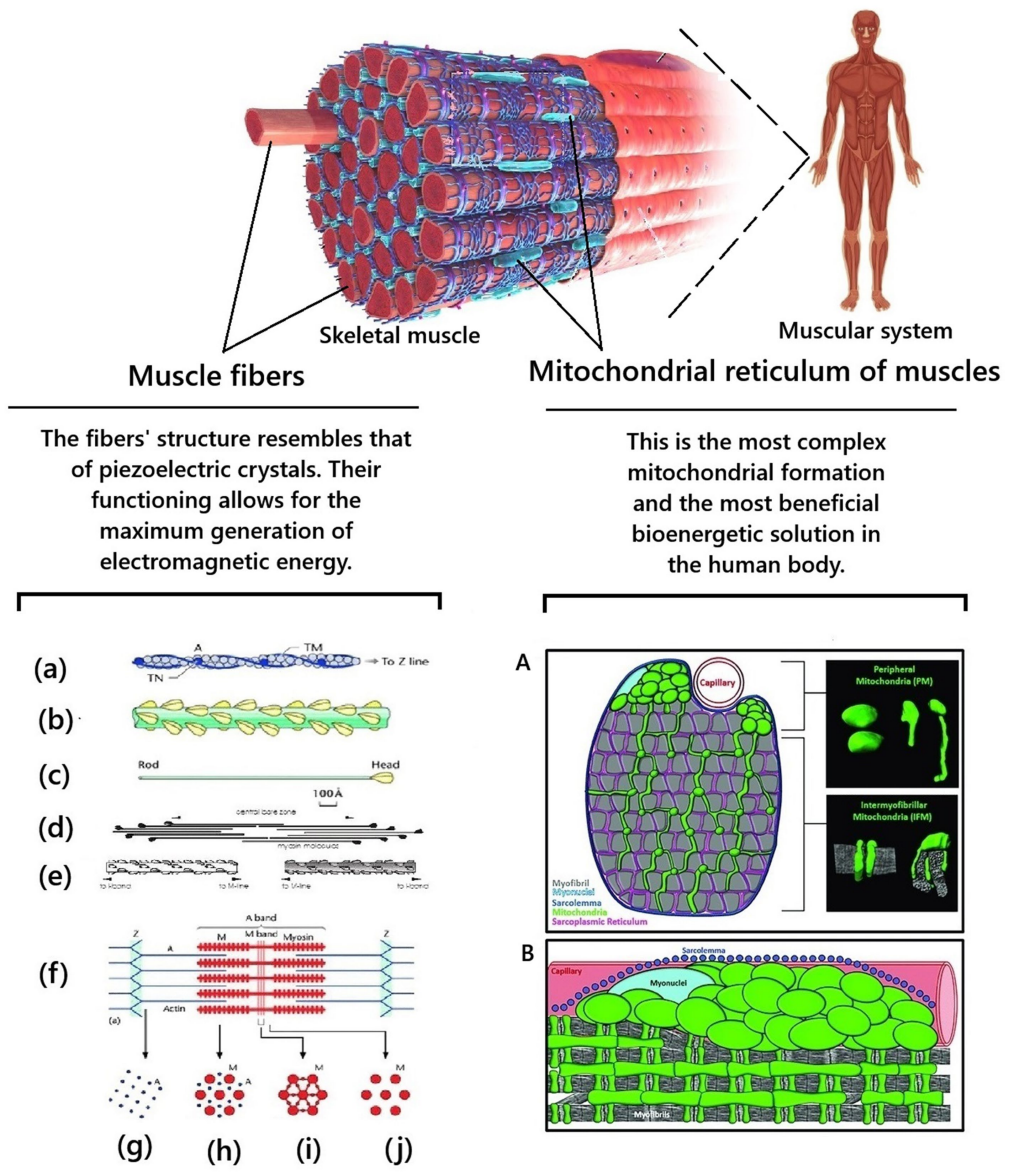
Biological features of the structure of skeletal muscles provide unique conditions for generating electromagnetic energy in the human body. Fragments from Squire (2016) and Willingham et al. (2021) were used. **(a)** Actin filament composed of actin molecules, (**A**) two tropomyosin strands, TM, and troponin molecule complexes, TN. **(b)** The bridge region of the myosin filament is composed of myosin molecules, shown in **(c)**, with the rod of the myosin molecules forming the backbone of the filament and the myosin heads arranged on the surface of the filament backbone. **(d)** The bipolar packing of the myosin molecules shows the anti-parallel arrangement, giving rise to a heads-free bare zone region at the center of the filament. This is also illustrated in **(e)**. **(f)** Sarcomere structure extending between two successive Z-bands, M: Myosin, A: Actin. **(g–j)** Cross-sectional views through different parts of the sarcomere, showing **(g)** the square lattice of actin filaments in the I-band, **(h)** the hexagonal lattice between overlapping arrays of actin and myosin filaments in the A-band, **(i,j)** the hexagonal lattice of myosin filaments in the M-band **(i)** and bare-zone **(j)** regions, with the extra M-protein density linking the myosin filaments at the M-region in the centre of the sarcomere **(i)** (from Squire, 2016). **A** is schematic cross-section of a muscle cell showing morphologically distinct subpopulations of peripherally located mitochondria and intermyofibrillar mitochondria. Mitochondria are highlighted in green. Myofibrils are highlighted in gray. The insets on the right are examples of peripheral mitochondria’s and intermyofibril mitochondria’s three-dimensional morphology. **B** is a longitudinal view of a muscle cell showing the subcellular arrangement of peripheral mitochondria and intermyofibril mitochondria relative to the sarcolemma (blue), capillaries (red), myonuclei (light blue), and myofibrils (gray) (from Willingham et al., 2021).

The brain has a maximum mass of up to 1.5 kg (Hartmann et al., 1994). This is less than 2% of the body weight of an adult. Striated muscles should typically comprise 40–50% of body weight, depending on age and gender (Alway et al., 2023). An adult male weighing 85 kg should have about 40 kg of muscle. Thus, normally (subject to proper physical development and an adequate level of physical activity of a person), muscles are the quantitative leader of the total content of mitochondria in the human body and the production of electromagnetic energy/biophoton signaling. It becomes clear that supporting and moving the body in space is not the only function of muscles. The results of a systematic analysis of existing scientific data have established that muscles are the final link in energy metabolism in the human body. The energy of chemical bonds of ATP molecules enters the biopolymers of muscle cells. They convert it into electromagnetic energy and other types of energy (mechanical energy of movement, thermal and acoustic energy, etc.). Thus, another important biological role of muscles in the human body is quantum electromagnetic energy generation for the whole body’s needs (Nevoit et al., 2024d, 2025a). Why is this so? The facts of the peculiarities of the histological structure of striated muscles confirm the correctness. Nature does nothing for no reason. Therefore, at the quantum level, the functioning of muscle cells is an example of how biophotonic mechanisms are integrated into the human body (Figure 3).

First, muscle fibers have a unique histological structure and organization at the quantum level. This is the basis for providing the most significant opportunity for generating electromagnetic energy by muscle cells at the quantum level. For example, actin and myosin molecules are located and packed with such precision that they approach the regularity of piezoelectric crystals. This creates conditions for significant generation of electromagnetic energy by biopolymers of the membrane structures of myocytes. At the moment of muscle contraction, electron tunneling (passage under the energy barrier, which occurs within a nanosecond) occurs, which is coordinated by fluctuations. Myocyte contraction occurs in specific and synchronous quantum steps. A typical muscle contraction involves a large number of myocytes, which perform the same milling of molecular filaments in a coordinated manner on a distance scale covering nine orders of magnitude. This ensures a chain-fluctuation-free process of actin-myosin filament sliding. Due to this, at the subcellular and deeper levels (quantum), due to the constant contraction of muscles, processes of transformation of the chemical energy of ATP into coherent electromagnetic energy occur (Ho, 2003; Nevoit et al., 2023; Nevoit et al., 2025a).

For future research in this scientific field, it is important to note that this coherent energy is formed under the influence of the biophotonic morphogenetic electromagnetic information field from the nucleus and mitochondrial DNA molecules. It takes over information from it and becomes its electromagnetic carrier during its further transport during cellular and transcellular communication in the human body. Therefore, electromagnetic currents of the human body are systemic information processes and should be studied in the future. This is already partially happening in science. For example, frequency-wave spectra are studied for the functional assessment of the functioning of the heart and the functional state of the whole organism in cardiology in the method of analyzing a short recording of heart rate variability (Eller, 2007; Nevoit et al., 2020), etc.

Secondly, muscle is the only tissue in the human body in which mitochondria are constantly organized into a single system – the mitochondrial reticulum (Glancy et al., 2015; Ryan, 2019). The mitochondrial reticulum is integrated into striated muscle cells in such a unique way that they essentially act as “quantum factories for generating electromagnetic energy.” Skeletal muscles consist of symplasts of a large number of myofibrils. Myofibrils contract synchronously and require a synchronous supply of large amounts of ATP. Myofibril mitochondria form a single three-dimensional system—the mitochondrial reticulum to meet this biological need. The mitochondrial reticulum permeates the entire muscle fiber: mitochondrial reticulum branches surround each myofibril in the muscle fiber; each sarcomere has two layers of mitochondrial reticulum; each muscle fiber has up to a thousand layers of mitochondrial reticulum; different layers are connected by mitochondria, which have a thread-like shape; adjacent branches of mitochondria are connected by mitochondrial contacts/junctions. The mitochondrial reticulum is a single cooperative energy system. The mitochondrial reticulum is the most advantageous bioenergetic solution. Each point on the inner surface of its mitochondrial membrane is equipotential: the electrochemical proton gradient is distributed uniformly in it and ATP synthesis can occur at any point (Kirkwood et al., 1986; Glancy et al., 2015; Gouspillou and Hepple, 2016; Campbell and Maani, 2023; Dong and Tsai, 2023). The myofibril mitochondrial reticulum is a unique “technical solution” and the most complex mitochondrial formation in the human body. Due to this, the cells of the striated muscles can instantly and coordinately produce a large amount of electromagnetic energy, which significantly exceeds the local needs of muscle tissue for it. This is why in Figure 2, muscles are shown as the main generator of electromagnetic energy/biophoton signaling.

**Figure 4:**
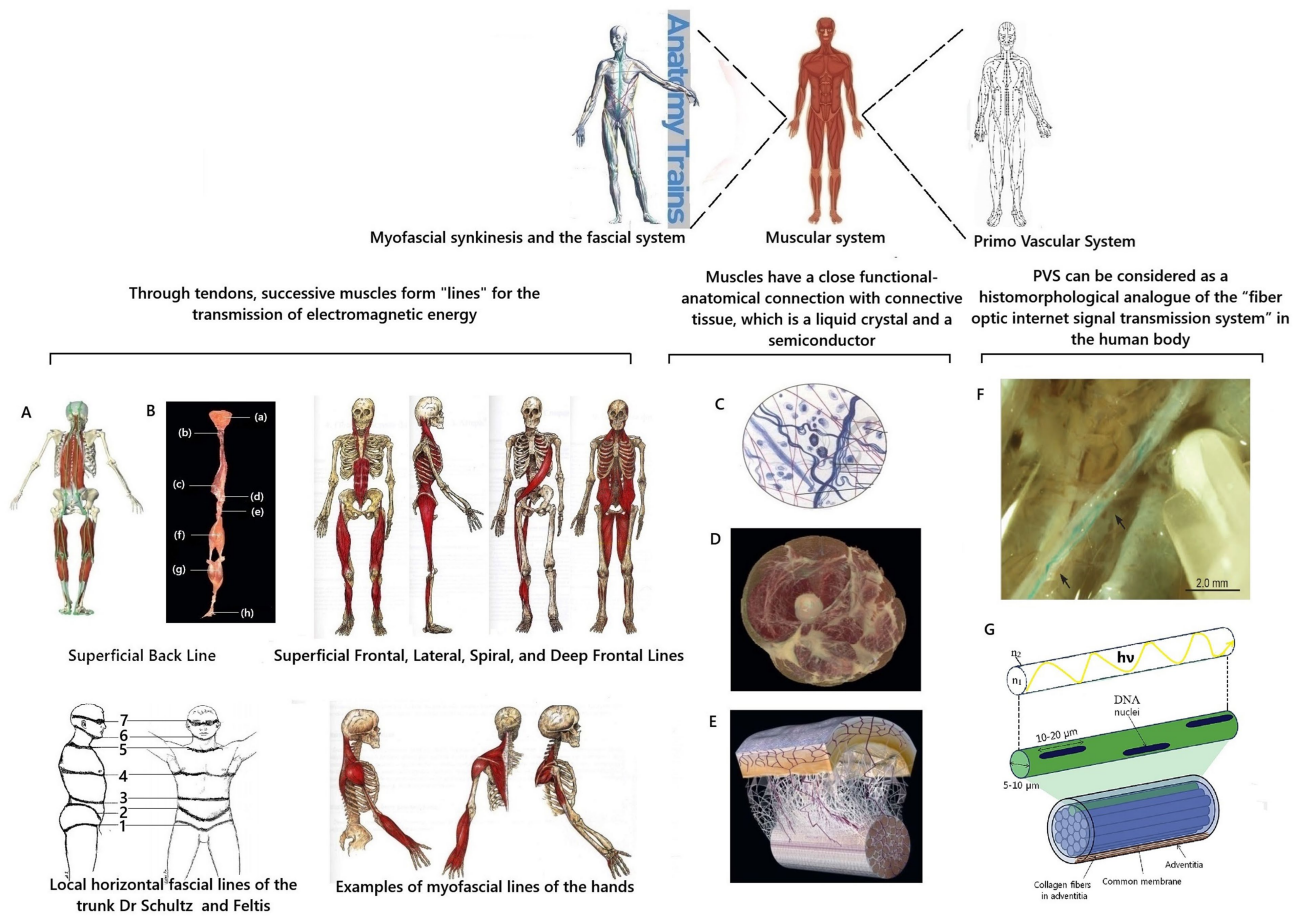
Scheme of substantiation of participation of the myofascial system and PVS in transmission of coherent electromagnetic energy in the human body. Fragments from Stefanov et al. (2013), Chikly et al. (2016), and Myers (2020) were used. **A** is Muscle synkenesis of the Superficial Posterior Line; **B** is dissected components of the Superficial Posterior Line (Myers, 2020); **(a)** epicranial fascia; **(b)** semispinalis capitis cervicis; **(c)** ilioclavicularis muscle; **(d)** sacral fascia; **(e)** sacrotuberous ligament; **(f)** hamstrings; **(g)** gastrocnemius muscle; **(h)** plantar aponeurosis; **C** is a diagram of the structure of connective tissue; **D** is a cross-section of human thigh demonstrating integration of fascia into muscle layers according to Myers (2020); **E** is a diagram of the fascial microvacuolar sliding system between the skin and underlying tendon according to Myers (2020); **F** is an *in situ* stereomicroscopic image of a PVS vessel in the thoracic duct of a rat according to K.-S. Soh. Arrows indicate areas of branching and reconnection (Chikly et al., 2016); **G** illustrates one isolated subvessel (top) and a bundle of subvessels of the primary vessel (Stefanov et al., 2013) with supplement. hν and the curved yellow line are schematic representations of the biophoton signal; n_1_ is the refractive index of the core; n_2_ is the refractive index of the cladding.

It is well known, understandable, and logical that the “excess” of electromagnetic energy in one place must move to another. Energy and information in the human body cannot be transmitted radiatively because it has a liquid crystal structure. Under what conditions can the transmission of coherent electromagnetic energy in the body of a living person occur with the participation of biophysical mechanisms and biological structures? The processes in the human body are subject to the universal laws of nature. Therefore, there must be biological structures in the human body to transmit energy and information between its parts. They should function like power lines and Internet communications. This is instead conditional, but it conveys the principle of the idea. Candidates for this role are the myofascial system of the human body and the Primo Vascular System (PVS) (Figure 4).

#### 4.2.1 The role of the myofascial system in the transmission of electromagnetic energy in the human body

In the human body, the myofascial system is precisely such a “system of cables” for transmitting coherent electromagnetic energy and information at the organismic level. On the one hand, the connective tissue of the fascia and tendons has a liquid crystal structure and is a semiconductor capable of conducting electromagnetic currents. These unique quantum mechanical characteristics of connective tissue explain the fact that it is now considered from a biophysical standpoint as an integral communication system in the human body (Oschman, 2000; Nevoit et al., 2024d, 2025a).

On the other hand, the correctness of this concept is confirmed by the peculiarities of the anatomical arrangement of muscles, ligaments, and fascia in the human body. Muscles are sequentially connected through tendons and fascia. They form muscle synkinesis/muscle chains or “muscle trains” (Myers, 2020). It turns out that the muscle contracts and generates specific coherent electromagnetic energy, which can be transmitted along the trajectory of myofascial synkinesis due to the semiconductor properties of connective tissue (Figures 4A–E). It is proposed to distinguish twelve myofascial synkinesis/muscle chains in the human body (Myers, 2020). They are located along the following lines (Myers, 2020): superficial posterior line, superficial frontal line, lateral line, spiral line, deep frontal line, and arm lines. All these lines are paired due to the bilateral symmetry of the human body. Due to tendons and fascia, these muscle chains are looped into one system, and their topography is close to the schematic arrangement of the so-called energy channels/energy meridians of ancient oriental medicine (Schlebusch et al., 2005; Stecco, 2015; Adstrum et al., 2017; Kirchgesner et al., 2018).

Research into the fascial system of the human body continues. For example, local horizontal fascial lines in the form of stripes on the body have been described (Schultz and Feltis, 1996). Further study of the features of the myofascial structure is important for further clarification and understanding of the biological role of fascia in the transmission of coherent electromagnetic energy/biophoton signaling between various organs and parts of the human body.

#### 4.2.2 The role of the PVS in the transmission of electromagnetic energy in the human body

**Figure 5:**
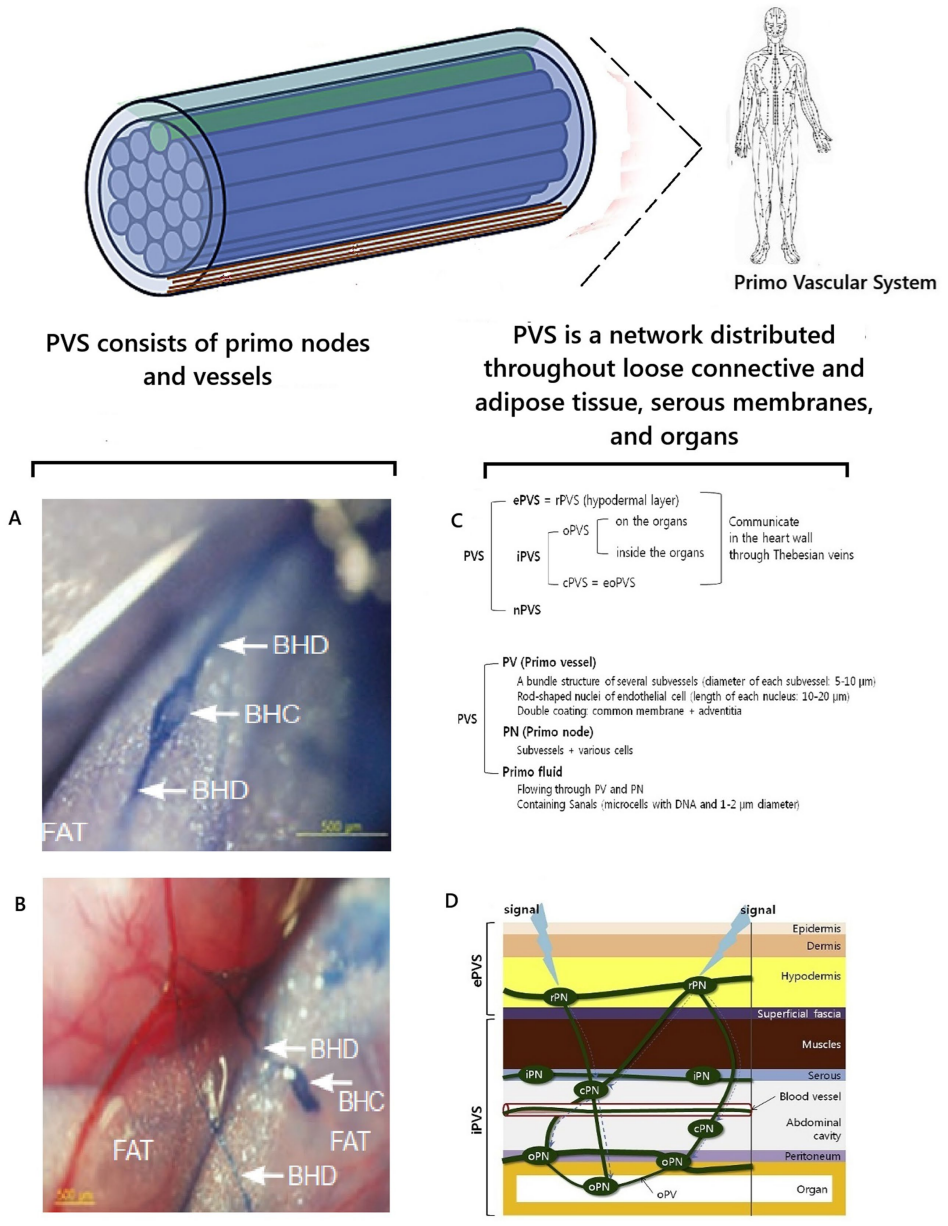
Visualization and localization of PVS. Illustrations were used (Stefanov et al., 2013; Chikly et al., 2016). **A,B** are preparations of adipose tissue near the small intestine of a rat, stained with trypan blue and visualizing PVS according to K.-S. Soh; BHD is Bong Han duct/vessel of PVS; BHC is Bong Han corpuscle/primo node (Chikly et al., 2016); **С** is an illustration of one isolated subvessel (top) and a bundle of subvessels of the primo vessel (Stefanov et al., 2013); **D** is the topographical distribution of the PVS (Stefanov et al., 2013); ePVS is the external subsystem; iPVS is the internal subsystem; nPVS is the nervous subsystem.

Metabolic information of biophoton signaling is strategically important for correctly coordinating the functioning of the human body. Therefore, nature could have created additional mechanisms for the protected rapid transmission of informational biophoton signals in the human body. A candidate for this role is the primary vascular system (PVS). PVS is a histomorphological analogue of the “fiber-optic Internet signal transmission system” in the human body. The existence of PVS was first discussed in science in 1960, when its discovery was announced by the Korean scientist Bong-Han (1962, 1963a, 1963b, 1964, 1965a, 1965b, 1965c, 1965d). Due to the high scientific controversy, knowledge about PVS was not integrated into orthodox science. This was due to the complete absence of ideas about biophotons in the scientific paradigm at that time. Also, Kim Bong-Han used a complex research methodology and a blue dye unknown to science, which he did not fully describe in his works. Therefore, other researchers were unable to repeat the results of his research (Stefanov et al., 2013; Chikly et al., 2016), casting doubt on his discovery. At the beginning of the 21st century, the existence of PVS as a new anatomical system of the human body and a morphological substrate of the ancient eastern energy meridian system was proven from the standpoint of evidence-based science by the scientific group of Soh (2004, 2009) and Soh et al. (2011, 2013). The features of the histomorphological structure of the PVS have been described in detail, and the corresponding terminology has been developed (Soh et al., 2011, 2013; Stefanov, 2012, 2022; Stefanov et al., 2013; Stefanov and Kim, 2015). It was found that the PVS consists of vessels and nodes and is distributed as a network throughout the loose connective tissue, adipose tissue, serous membranes, and organ tissues. Depending on the localization, it was proposed to classify the PVS into external, internal, and nervous subsystems. The external PVS subsystem is localized in the skin’s hypodermal layer and the superficial fascia. The internal PVS subsystem is inside the blood and lymphatic vessels, heart chambers, and organs. The nervous subsystem of the PVS is located in the brain’s cavities, in the spinal cord canal, and is connected with the epinervium and perinervium of the nerves (Stefanov et al., 2013) (Figure 5).

At the same time, the hypothesis was first put forward that PVS is involved in regulating the body and acts as an optical channel of biophoton emission (Stefanov et al., 2013). What facts substantiate this idea? According to the conducted system analysis of the existing scientific data, it was found that the histomorphological structure of PVS vessels is indeed fundamentally similar to the structure of optical fiber (Figures 5F,G). The PVS vessel and optical fiber have a two-layer structure and consist of a “core” and a shell with different refractive indices n1 and n2. The core is the central region through which the main part of the optical power of the signal is transmitted. This occurs because the refractive index of the shell is much lower than that of the core. Therefore, light/biophotons are reflected at the “core-shell” boundary. As a result, the signal moves forward in the optically denser medium of the core, without going beyond its limits. In optical fibers, the dielectric material for the core is fused quartz (quartz glass), and on the outside, there is a shell and a protective coating to protect against mechanical impacts. In PVS, this function is performed by primo-liquid, which flows in primo vessels. It has a unique, specific composition, fundamentally different from the composition of lymph and blood. This is a transparent liquid that has a complex composition and contains free nucleic acids—DNA, adrenaline, noradrenaline, stem cells (analyses according to Kim Bong-Han), granulocytes, mast cells, histiocytes, eosinophils, neutrophils, and single lymphocytes (Sung et al., 2010; Chikly et al., 2016).

Like optical fiber, PVS vessels have a shell to protect against mechanical and physical impacts. PVS vessels have a double shell. Each subvessel has a common membrane. The entire vessel is surrounded by adventitia, which contains collagen fibers as a supporting and protective component (Soh, 2009). The collagen of the shell prevents the penetration of photon radiation from other external biomolecular sources (Stefanov, 2012) and helps to “protect” the biophoton signal. This additionally ensures the tuning of photon radiation throughout the body (Stefanov et al., 2013). Thus, the histomorphological principle of the structure of PVS vessels is fundamentally similar to the principle of the optical fiber. This allows us to conclude that the biological purpose of PVS is a biological complex of channels for transmitting optical signals in the human body. The term “optical signal” should be understood as standing waves of coherent electromagnetic energy formed by biopolymers of cell membranes with information content from cell DNA molecules—the previously described phenomenon of biophoton signaling.

During the study of the composition of the PVS liquid, a significant, unique fact was discovered. It contained free DNA molecules (Ogay et al., 2006; Stefanov et al., 2013). The biological purpose of these DNA molecules was unclear because the processes of division and synthesis, according to the scientific paradigm, could not occur in these vessels. Now, this fact can be scientifically explained from the position of the concept of biophoton signaling and knowledge of the existence of electromagnetic mechanisms of DNA functioning. Since DNA molecules can accumulate electromagnetic energy and emit it like a laser, DNA molecules in the PVS liquid can function as retrosensors. Their biophoton radiation can compensate for the attenuation of the optical/biophoton signal, correct its distortions, and thereby increase its noise immunity when passing through the PVS vessels.

PVS studies have established the fact that the bioelectric signals of PVS endothelial cells are similar to the signals of smooth muscle cells, and PVS has bioelectric activity, excitatory conductivity, and mechanical mobility (Park, 2009; Jia et al., 2010; Stefanov et al., 2013). It was established that the functional state of the PVS changes depending on the changing needs of the organ tissue. When systemic/general organ reactions occurred, activation of the “dormant” parts of the PVS was noted (Stefanov et al., 2013). The PVS response occurred to mechanical and electrical stimuli (Stefanov et al., 2013). It was found that the PVS vessels have excitable nerve-like structures and exhibit spontaneous electrical activity. When measuring electrical conductivity, it was found that three periodic potentials were characteristic of the PVS vessels, lasting from 15 to 30 s, from 7 to 10 s, and from 20 to 25 s (Chikly et al., 2016). This indicates that the PVS is included in the communication system of connections between the body’s cells. It was assumed that, under pathological influence, damaged cells send signals, and PVS transmits them from the site of damage further to the cells of other tissues and organs, ensuring the flow of electromagnetic energy and information to them, which is necessary for their restoration.

Another indirect confirmation of the biological role of PVS as a morphological substrate for the translation of biophoton signaling is the established biological fact that PVS is formed earlier than extraembryonic vessels, the heart, and intramembrane vessels (Lee et al., 2023). After the PVS has formed, the vascular and nervous systems are formed and developed around it. From the biophoton signaling concept standpoint, this is explained by the fact that the PVS creates an “electromagnetic framework” /morphogenetic electromagnetic field for tissue development. This explains the close anatomical and functional connection between the nervous, vascular, and endocrine systems and the PVS installed in the human body. It also becomes clear that such “accompaniment” of the PVS of the nervous, vascular, and endocrine systems is not accidental from a biological point of view. This is a way to ensure electromagnetic communication and control of the functions of these systems (Stefanov et al., 2013) through biophoton signaling. This gives additional grounds to consider the idea correct that the PVS, as a biophoton transport system, is involved in transmitting electromagnetic energy and information between cells, and is needed to form electromagnetic fields during the ontogenesis of the human body.

Integrating of this knowledge into the existing scientific paradigm continues (Vodyanoy et al., 2015). Further study of the role of biophotons in the human body and the creation of the concept of biophoton signaling are other scientific steps towards this. Histomorphological features of the structure of PVS have been studied in detail (Stefanov, 2012, 2022; Stefanov et al., 2013; Stefanov and Kim, 2015). PVS has such a histological structure that it allows for the transmission of biophoton signaling energy and information uniquely throughout the body. This is a different and fundamentally new approach for the modern scientific paradigm, which requires further conceptualization in the future. The fact of the proven morphological existence of PVS, the coincidence of PVS channels with the trajectory of the meridian system of ancient Eastern medicine and with muscle synkinesis confirms the correctness of the concept of the important quantum role of the participation of striated muscles in the processes of generation and transmission of the biophoton signal in the human body. The concept of biophoton signaling may help resolve one of the paradoxes of modern science (Nevoit et al., 2024d). A paradoxical situation has developed around scientific knowledge about PVS: despite being fully scientifically proven, the results of these studies are not integrated into fundamental science and do not receive recognition and popularization (Stefanov, 2022; Nevoit et al., 2024d).

The correctness of the theory is consistently confirmed by practice. In this case, the correctness of the presented concept is evidenced by the proven experience of the positive practical use of reflexotherapy methods in treating diseases of the human body (Tsuchida and Kobayashi, 2020; Runge et al., 2022). The presented concept of biophoton signaling is another active mechanism of reflexotherapy (van Wijk et al., 2010; Bordoni et al., 2018; Nevoit et al., 2024c).

### 4.3 Biophoton signaling in the brain

The brain is the most important organ of the human body, ensuring its higher nervous activity and coordinating all metabolic and functional processes. Therefore, the development of aspects of biophoton signaling in the brain is an important part of the concept. This is a new and important scientific aspect that requires further study.

The brain’s functioning is carried out due to the generation of coherent electromagnetic energy by biopolymers of its cell membranes and the subsequent unique interaction of these resulting electromagnetic currents/biophoton signals with each other. This is based on unique mechanisms of transmission/redistribution of biophoton signals in the tissues of all brain areas. Ultimately, this electromagnetic cellular activity creates a unique local electromagnetic field of the human brain, ensures the functioning of the human body, and its life as a rational being.

According to the universal laws of physics, electromagnetic fields must interact. Therefore, it is logical to believe that electromagnetic energy from the brain should somehow replenish the human body’s electromagnetic field, formed by the activity of its other tissues and organs. The brain’s electromagnetic field is an important integral component of biophoton signaling and a source of information for the general electromagnetic field of the human body. The study of quantum mechanisms of interaction of electromagnetic fields and biophoton signaling in various areas of the brain is an important and promising direction for future neuroscience development. This is a new scientific direction that is just emerging. How can existing scientific knowledge about the structure and functioning of the brain be integrated into the concept of biophoton signaling?

**Figure 6:**
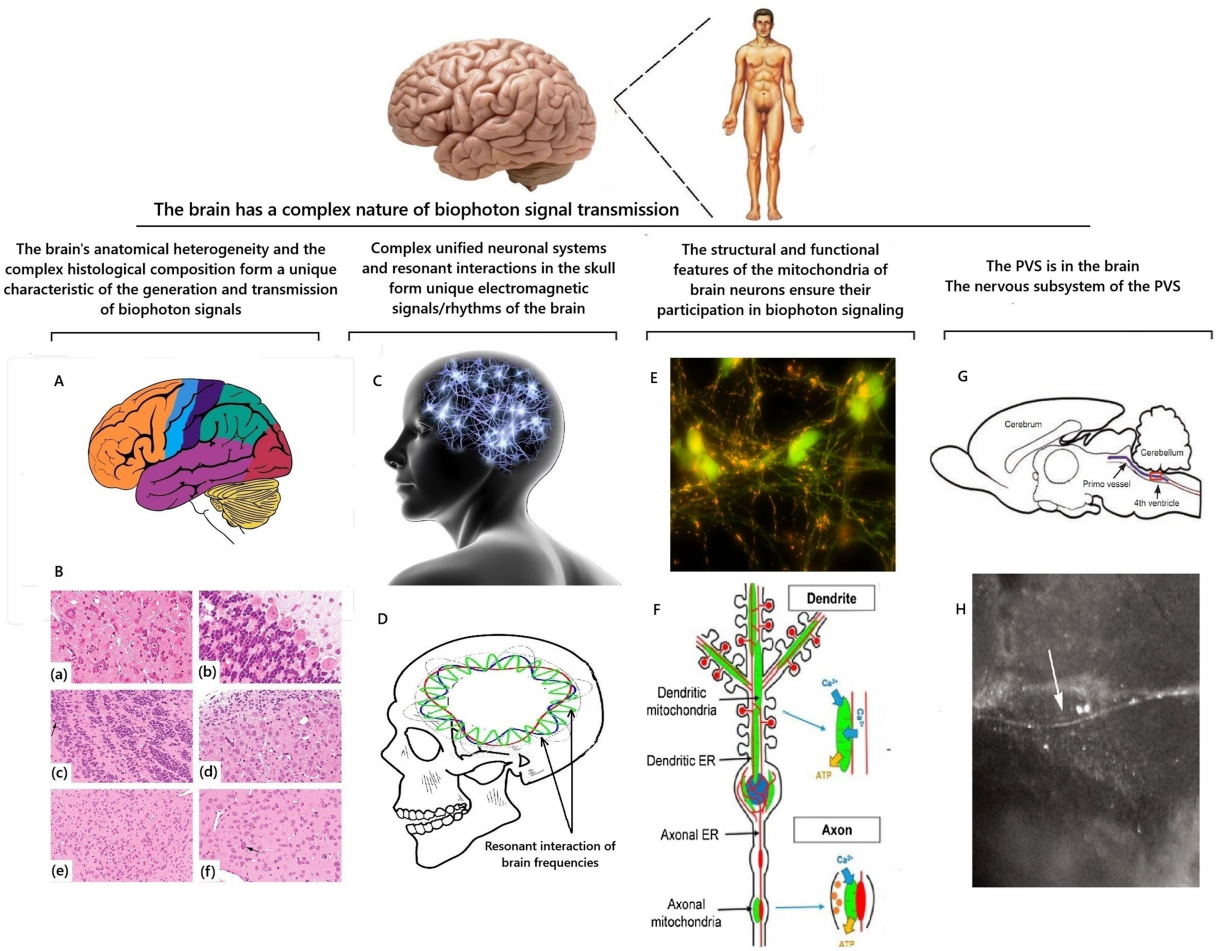
Scheme of factors that determine the characteristics of the propagation of a biophotonic signal inside the brain. Illustrations are based on Garman (2010), Chikly et al. (2016), Stępkowski et al. (2017), and Rangaraju et al. (2019). **A** is an anatomical division of the brain into zones; **B** is an example of heterogeneity of neuronal populations in the brain on sections stained with hematoxylin and eosin; final magnifications: **(a,c,d)** = 277x; **(b)** = 554x; € = 138x; **(a)** there is the reticular formation formed by medium-to large-sized neurons with prominent Nissl substance; **(b)** there is the cerebellum and **(c)** there is the olfactory bulb, which are composed of a single layer of large projection neurons (Purkinje neurons for the cerebellum and mitral cells (arrows) for the olfactory bulb) and a large number of small interneurons; **(d)** there is the cochlear nucleus, which contains medium and large neurons together with a “cap” of granule cells; **(e)** there is the amygdala, which is formed by a relatively monomorphic population of medium-sized neurons; **(f)** there is the striatum (caudate nucleus-putamen), which is composed mainly of medium-sized neurons together with scattered large cholinergic interneurons (arrow) (Garman, 2010); **C** is a symbolic representation of the brain as a single neural network; **D** is a schematic representation of intracranial resonances of electromagnetic frequencies of the brain; **E** is a photograph of mitochondria (multiple yellow inclusions) in neurons (Stępkowski et al., 2017); **F** is a diagram of the arrangement of mitochondria in a neuron (Rangaraju et al., 2019); **G** is an illustration and iof the primo vessel (arrows) floating inside the ventricular system of the brain by Chikly et al. (2016); **H** is an image of the primo vessel (arrows) floating inside the brain’s ventricular system by Chikly et al. (2016).

The principle of the structure of brain cells is analogous to all other cells of the human body. Therefore, at the micro level, the processes of biophoton signaling in brain cells should occur similarly to those described for other body cells. However, the unique internal structure of the brain will provide a completely different and still not fully explained from a scientific point of view nature of the propagation of the biophoton signal inside the brain and the quantum interaction of these signals with each other (Figure 6).

#### 4.3.1 Brain histomorphology and biophoton signaling

The uniqueness of biophoton signaling of the adult human brain is formed due to the complexity of the histomorphological structure and functions of its different areas. The brain has a complex histological composition of many neurons and neuroglial cells (Garman, 2010). Therefore, all areas of the brain have significant features of individual structure, fundamental histological differences from each other, and different quantum-mechanical characteristics of the molecular components of cells (Figures 6A,B). Because of this, their contribution to the overall electromagnetic generation will be quantitatively and qualitatively different. This will depend on the individual features of the structure and functional state of brain cells formed during a specific individual’s ontogenesis.

#### 4.3.2 Brain neural networks and biophotonic signaling

The brain contains 20,000 neurons per mm^3^ (von Bartheld et al., 2016). The shape and quantum-mechanical composition of neurons and their membrane structures are unique regarding the possibilities of electromagnetic communication between cells. Long processes/axons in neurons ensure the transfer of electrical and magnetic signals over long distances from the neuron’s body. Short processes/dendrites form complex interlocking neuronal connections between neurons. Each neuron has 40,000 synapses (Panov et al., 2023). This unit integrates all the brain’s cells into a single complex system of electromagnetic generation (Figure 6C). Neurons of each zone generate a coherent electromagnetic signal, exchange it through synapses, and create individual frequency-wave patterns. These patterns form local electromagnetic fields, which are more pronounced in the zones of current functional activation of the brain. These coherent electromagnetic fields of brain cells cause frequency-wave processes specific for each zone. These electromagnetic wave processes interact in the brain according to the Field Theory. Since the skull is a resonator, specific conditions are created for damping and amplifying the waves of electromagnetic generation from brain cells. The result of this interaction is the occurrence of characteristic yogic resonant frequencies of the brain, which are called rhythms of the brain (Figure 5D). Brain rhythms coincide with the so-called Schumann resonance frequencies of the Earth and are essentially an analogous coherent electromagnetic process with a soliton mechanism (Salari et al., 2022; Nevoit et al., 2025b). The existence of a complex unified system of neuronal electromagnetic generation and its unique interaction in the human cranial cavity determines that the brain can be functionally considered as a coherent system. This is reflected in the studies of many scientists (Rahnama et al., 2011; Salari et al., 2011, 2012; Cacha and Poznanski, 2014; Moradi et al., 2015). The results of electroencephalographic recording of coherent changes in large cellular clusters, even in remote brain areas, confirm this (Salari et al., 2015). Thus, a multicomponent three-dimensional organization in space of a large number of brain cells with different functions forms a complex system of conjugation of electromagnetic fields. Creating a private concept of the features of the distribution and transmission of a biophotonic signal in the brain is a challenge for modern scientists and a task for future theoretical research.

#### 4.3.3 Features of neuronal mitochondria and biophoton signaling

Brain cells differ in the number, location, and shape of mitochondria. In the human body, neurons of the brain are leaders in mitochondrial content, as are cells of striated muscles (Bruce and Johnson, 2002). Since neurons have a complex structure with multiple processes, the shape and function of neuronal mitochondria differ in different parts of the neuron (Figure 6F). In the neuron body, mitochondria merge, forming a dense network. The mitochondrial network is the most advantageous bioenergetic solution and, in all likelihood, it is appropriate in the neuron body, where the main metabolic processes and the formation of new mitochondria occur. Newly formed mitochondria migrate into their processes towards the synapses. In dendrites, mitochondria have a strongly elongated shape and resemble spaghetti. Dendrites have many synapses and a narrow, elongated shape. Therefore, the shape of mitochondria repeats itself. In axons, mitochondria have a shorter or close to spherical shape (Rangaraju et al., 2019; Panov et al., 2023). Axons are longer. Therefore, the shape and size of mitochondria are convenient for the constant migration of mitochondria along microtubules along the axon (Figure 6E). This is demonstrated by the video recording of Stępkowski et al. (2017). All this causes pronounced differences in biophoton signaling from those described in muscle cells, allowing us to assume a different and more complex nature of biophoton signal translation.

As noted earlier, the entire logic of the biophoton signaling concept is valid for all brain cells. In neurons, the basic information biophoton signal comes from the DNA molecules of the nucleus. Then, biophoton signaling from the DNA of the nucleus of neurons goes to a “powerful electromagnetic carrier,” which is an electromagnetic field created by oscillations of the biopolymers of the membrane structures of neurons. This fills and modulates the information component of the electromagnetic field of the corresponding areas of the brain. Mitochondria of neurons also generate biophotons. Apparently, due to the large number of mitochondria and the microtubule system, the problem of creating the necessary signal density parameters in all neuron areas is solved. This is confirmed by the topical location of mitochondria in neurons and their shape. Dendrites are almost filled with mitochondria that repeat their shape, and spherical mitochondria constantly migrate in axons (Rangaraju et al., 2019; Panov et al., 2023). Thus, conditions for adequate maintenance of biophoton signaling and its density are formed throughout the entire volume of the neuron body and in its processes due to the activity of mitochondrial DNA. A significant accumulation of mitochondria is always present in the synapse area (Panov et al., 2023). Previously, this was explained by significant energy needs in this zone. However, there is evidence that neuronal mitochondria are not oriented towards synthetic processes and do not have endogenous reserves of substrates. These processes are provided by the mitochondria of astrocytes (Panov et al., 2023). Another biological concept, biophoton signaling, can explain this. In synapses, coherent electromagnetic energy/biophoton signal is constantly transmitted from one neuron to another. Because of this, biophoton signaling needs to be enhanced precisely in the zone of interneuronal interactions. This is necessary for the implementation of information and energy exchange in synapses. Moreover, this task is solved by mitochondrial DNA, the clusters of mitochondria present there.

The energy and information of biophoton signaling circulate through the brain’s system of neuronal connections. It can also be assumed that the processes of biophoton signaling circulation should be more intense in those areas of the brain that a person uses at this moment in their life. This logically fits into the existing research results on the brain’s electromagnetic activity and does not contradict the modern paradigm.

#### 4.3.4 Participation of the PVS nervous subsystem in biophoton signaling

According to the results of researchers (Bong-Han, 1962, 1963a, 1963b, 1964, 1965a, 1965b, 1965c, 1965d; Soh, 2004, 2009; Soh et al., 2011, 2013; Stefanov et al., 2013; Chikly et al., 2016), who studied the PVS, this system is in the brain. This part of the PVS is called the nervous subsystem. The brain is the central organ of the nervous subsystem (Figures 6G,H). The vessels of the nervous subsystem PVS are located in the cavity and the membranes of the brain, in the spinal canal, and along the nerves. The PVS nodes are localized in the membranes of the brain (meninges) and the brain (Bong-Han, 1965b). Various parts of the PVS nervous subsystem communicate via sinuses and cisterns of the dura mater. Thus, it is possible to transmit electromagnetic signals/biophoton signaling from the brain to the tissues of organs and back. The existence of a connection through the PVS between the coherent electromagnetic field of the brain and organs is a scientific explanation of the mechanisms of occurrence of psychosomatic disorders. Two-way interaction between the brain and organs via biophoton signaling can explain the phenomenon of the influence of human thoughts on the functional state of the body and vice versa. Detailing the quantum mechanisms of how this happens is a challenge for neuroscience scientists of the future.

#### 4.3.5 Prospects for research into biophotonic signaling in the brain

Of course, all these are new ideas for creating new directions of scientific research. In the future, all accumulated scientific material regarding the existence and structural aspects of PVS should be subjected to additional system analysis. The results of such studies will resolve the issue of further full integration of knowledge about PVS into the system of medical sciences. Neurons are intricately looped among themselves in the multi-hierarchical, heterogeneous structural zonality of the brain. Therefore, in the near future, it will be difficult to create even a promising model of the distribution map of electromagnetic fields and biophoton signaling in the brain. But nothing is impossible. A hundred years ago, the results of Gurvich’s research (Gurwitsch, 1911, 1923, 1924; Gurwitsch and Gurwitsch, 1960) were perceived as incomprehensible and impossible. Now they seem simple and logical. Therefore, science should simply move forward, and the secret of biophoton signaling of the brain will be unraveled.

The analysis of magnetoencephalogram data can now become the basis for creating working hypotheses about the circulation of electromagnetic fields in different brain areas. This also requires a deeper understanding of the spectrum of frequencies emitted by brain cells and improving their clinical analysis in the future. Spectral analysis of electroencephalograms can be used for this purpose.

The most complex and still incomprehensible question for modern science is the mechanism of the emergence of higher nervous activity and consciousness. There is a scientific assumption that UPE, microtubules, and quantum biology contribute to or generate consciousness (Craddock et al., 2016). This is probably true, but it is too broad a generalization. The same conclusion can be made about the theory of orchestrated object reduction (Hameroff and Penrose, 2014). If we look critically at these assumptions, they are legitimate in creating quantum fields. Their imperfection lies elsewhere. Where does the information content come from, and for which concepts of “mind” or “ego” are used? It is more expedient to connect everything, relying on the emission of biophotons from DNA molecules. It can be assumed that such unique metabolic and electromagnetic field environments are created in the neurons of the brain, which awaken specific gene expression in DNA molecules. This unique, specific state of DNA gene expression causes a highly personalized, unique combination of biophoton emission from the DNA core of neurons, which throughout a person’s life fills the quantum electromagnetic field of his brain with the awareness of his “ego” and his “mind.” This occurs like the described process in somatic cells, in which biophoton signaling is “entered” as information on a coherent flow formed by the oscillation of biopolymers of membrane structures. The same thing happens in the brain. Only in the case of the brain is this flow of electromagnetic energy formed by the membrane structures of neurons, as described earlier. Why is DNA the source of information, and not microtubules? Let us remember where human life begins. It begins from the set of chromosome apparatus in one fertilized egg. All information is preserved in DNA structures.

A more logical explanation is that microtubules perform only the transport and consolidation function of the electromagnetic signal. Their role is similar to that of PVS and the myofascial system, which distributes electromagnetic energy throughout the body. This is consistent with studies showing that the arrangement of aromatic tubulin dimers that make up microtubules facilitates the diffusion of photoexcitation-induced electron energy over significant distances (Kalra et al., 2023). This effect was reduced and simulated in the presence of an anesthetic (Craddock et al., 2017). The arrangement of microtubules has been found to provide non-trivial quantum effects that are resistant to decoherence (Hameroff et al., 2002). It was also found that the microtubular cytoskeleton is close to mitochondria, a potent source of UPE, and can absorb and direct UPE-induced excitation over long distances via resonant energy transfer (Kurian et al., 2017). Thus, interactions between UPE and microtubules exist, but DNA molecules should still be considered the source of biophoton emission.

## 5 Discussion, problems and prospects

What is the scientific and practical value of the biophoton signaling concept described in the review?

The presented promising working concept of biophoton signaling continues the creation of a biological medical theory that could systematize and unite, on its basis, a multitude of multidirectional results of studies of electromagnetic parameters of the functioning of organs and organ systems in the human body—the Magnetoelectrochemical Theory of Metabolism and Life (Mintser et al., 2020, 2021, 2022, 2023; Nevoit et al., 2024b).

The concept of biophoton signaling deepens fundamental ideas about energy exchange in the human body. It clarifies the electromagnetic mechanisms of DNA information participation in the metabolism of human body cells. All this demonstrates the reality and importance of the electromagnetic component of cellular communication in the phenomenology of biological life at the micro level of the human body.

The presented concept of biophoton signaling makes it possible to answer the questions outlined in the introduction. Based on the presented data, it is clear that the human body is united into a single functional whole using biophoton signaling mechanisms. Biophotonic signaling from DNA molecules forms, together with coherent energy generated by biopolymers, the cells’ internal electromagnetic information field. This internal electromagnetic field of the cell creates conditions for constant information filling inside the cell without contact. It ensures instant coordination of the simultaneous flow of many metabolic reactions. This information field ensures the folding of proteins and other complex molecules, controls the morphogenesis of the cell, and all cycles of its development. The circulation of coherent electromagnetic energy in the form of solitons through the semiconductor structures of the cell (biopolymers, liquid crystals of structured water, lipids, connective tissue, etc.) supplies molecules with energy and information, revives them, and makes them part of a single functional whole. This is consistent with the findings that molecules with an aromatic ring (tryptophan, tyrosine) may be “detectors” or “receptors” for biophotons and are found in high density in the microtubules of the cytoskeleton (Craddock et al., 2014). Several scientists believe that it could be a multitude of other fundamental molecules with double-bond structures from which life evolved as a dissipative structure (Nunn et al., 2020) or even any biological molecule capable of absorbing a photon, changing the structure/pathway in which it is located (Mould et al., 2024). Also, the concept of biophoton signaling is consistent with the results of works that described models of excitation transmission/coherent “beating” caused by UPE along the length of the cytoskeleton with the location of tyrosine and tryptophan residues (McFadden and Al-Khalili, 2018) or involving mitochondria, mitochondrial networks and microtubules, which can act as “organic fiber optic networks” and provide high-speed communication throughout the cell (Thar and Kühl, 2004). In these studies, UPE was described as a highly plausible, rapid, and robust mechanism of intracellular communication to maintain cellular homeostasis. The energy transfer mechanisms in these models were described as similar to quantum photosynthesis models (McFadden and Al-Khalili, 2018). Linked to the ideas of biophoton signaling concepts, a study on superradiance (Celardo et al., 2019) describes a mechanism for collective radiation of the system that follows coherent excitation in microtubules. This results in an amplification of the photon signal by biological microtubule structures. This explains how cells can use these initially produced low levels of photons. This suggests that the signal inside the cell may be stronger than what is registered outside the biological organism. This is supported by the opinion of Bókkon et al. (2010).

The concept of biophoton signaling forms a new view and deepens the knowledge of NCDs’ pathogenesis. Biophoton signaling in the tissues of each organ has specific energetic and information components for this organ. It contributes to the general electromagnetic interaction of cells at the organism level. Qualitative and quantitative parameters of biophoton signaling are determined and depend on the functional state of the cell. Pathogenetic influence of epigenetic factors and DNA mutations will affect gene expression and information that enters the electromagnetic field of the cell through biophoton signaling. Mitochondria are key organelles that ensure adequate biophotonic signaling processes. The more mitochondrial DNA a cell has, the more sources of biophoton signaling there will be in the cell, and the greater the density of the electromagnetic field will be. A decrease in the number of mitochondria, mitochondrial dysfunction, degradation of the mitochondrial pool, and mutations of mitochondrial DNA can lead to pathological changes in the information component of biophoton signaling and a decrease in the power of electromagnetic generation in the cell. The amount of ATP formed and molecular synthesis in the cell depend on the activity level of the Krebs cycle and oxidative phosphorylation processes. A decrease in ATP synthesis will lead to a decrease in its supply to biopolymers. Therefore, cellular energy deficiency due to mitochondrial dysfunction is manifested by a decrease in electromagnetic generation by the membrane structures of the cell. Objectively, this can be registered as a decrease in the action potential of cytoplasmic membranes. Magnetometry of organs can also record a decrease in the corresponding indicators. Violating molecular synthesis processes due to mitochondrial dysfunction will change the molecular composition and quantum-mechanical characteristics of cellular structures. This may result in changes in their ability to resonate and receive the biophotonic signal. If pathological deviations in biophoton signaling and in the ability of cellular structures to adequately receive an information signal persist and progress, this will cause a gradual increase in pathological changes in metabolism in the cell and lead to an increase in pathohistomorphological changes. Further, increased changes in the “molecular landscape” of the cell and the quantum-mechanical characteristics of cellular structures will reduce their ability to perceive biophoton signaling information adequately. This will lead to a pathological circle and an ever-increasing progression of cell pathology. Therefore, the pathology of biophoton signaling is a new quantum mechanism of NCDs pathogenesis, which deserves due attention from medical science. NCDs are an important unsolved medical problem of public health (Mikkelsen et al., 2019; Kostova et al., 2021; Kundu and Chakraborty, 2023; Andrade et al., 2023), which already has the status of a pandemic (The Lancet, 2022). Therefore, the search for new mechanisms of NCDs pathogenesis is relevant. Deepening fundamental knowledge of NCDs pathogenesis will contribute to developing new approaches to solving this problem. Thus, understanding the essence of the mechanisms of electromagnetic cellular communication is a fundamentally new and promising direction for improving the scientific paradigm of NCDs in the aspect of quantum mechanisms of their pathogenesis.

The proposed working model of biophotonic signaling describes its basic mechanisms, which are universal for all cells, and fits it into the system of existing knowledge. However, there is more unknown to science than known. This can be seen in the example of examining specific aspects of biophoton signaling of different organs of the human body, and the brain, in particular. In the future, it is necessary to develop concepts for all human body organs. The topic of mechanisms of biophoton signaling in the brain is at an early stage of development, and it is necessary to continue research in this direction. Many scientists share this opinion (Mould et al., 2024). Certainly, biophoton signaling is involved in the functioning of the brain as a fundamental and universal mechanism for the distant transmission of energy and information. Biophoton signaling certainly provides control over metabolic and functional processes in the brain. This happens similarly to other cells in the human body.

The concept of biophoton signaling has brought orthodox science and traditional medicine closer together to a certain extent. Thanks to the concept of biophoton signaling, another scientifically substantiated opportunity has appeared to deepen the understanding of the mechanisms of clinical effectiveness of reflexology methods and several other traditional medicine approaches. This is in line with the findings of the World Health Organization (WHO) at the Traditional Medicine Global Summit 2023 meeting report: Gujarat Declaration. According to paragraph 12 of the Gujarat Declaration, science, technology, innovation, and knowledge sharing must be used to validate and promote traditional medicine. According to paragraph 14 of the Gujarat Declaration, it is also necessary to contribute to the development, implementation, monitoring, and transformative impact of the WHO Global Traditional Medicine Strategy for the period 2025–2034, and advocate for increased political and financial commitments at global, regional, national and community levels to translate that strategy into policies and practices for people’s health and well-being (WHO Traditional Medicine Global Summit, 2023).

The concept of biophoton signaling requires further development. On the one hand, this requires searching for and developing new methodological approaches to research. Modern technological progress ensures rapid development of technologies. Therefore, the number of technical possibilities for the registration of biophotons is increasing. Equipment that can be used in the study and its advantages and disadvantages are described in detail in the review by Mould et al. (2024). Mould et al. believe that the slow implementation of UPE research is due to difficulties in experimentation. This is true. However, on the other hand, scientific progress in this direction requires the willingness of the global academic community to support these new ideas in their creative development. An adequate level of scientific interest in this area of research will become the basis for a rapid paradigm shift. It will create favorable conditions for obtaining grant funding for this research and publishing research results in the future. A transdisciplinary approach is also needed. The development of the concept requires the participation of physicists, IT specialists, and biomedical specialists.

## 6 Conclusion

(1) Emission of biophotons, cellular electromagnetic communication/biophoton signaling, are objectively existing and scientifically proven phenomena of the biological life of cells. (2) Biophotonic signaling is a scientifically proven mechanism of electromagnetic communication of cells, which is of key importance at all stages of their life activity. (3) The proposed promising working model of biophotonic signaling is a scientific tool for further deepening fundamental knowledge about the functioning of the human body in normal and pathological conditions. This is the next step in understanding the quantum processes of energy and information transfer in the human body and developing the direction of Biophotonics. (4) Biophoton signaling is a new component of NCDs pathogenesis that requires further study. (5) Scientific discussion regarding the ideas of a promising working concept of biophotonic signaling and further development of the scientific direction on the role of electromagnetic signaling in the human body and brain should be continued.
